# Metabolic profile and bioactivity of the peel of Zhoupigan (*Citrus reticulata* cv. Manau Gan), a special citrus variety in China, based on GC–MS, UPLC-ESI-MS/MS analysis, and *in vitro* assay

**DOI:** 10.1016/j.fochx.2024.101719

**Published:** 2024-08-06

**Authors:** Jialiang Zou, Peng Wang, Huanhuan Xu, Xuelian Gan, Huangsheng Zhang, Lin Chen, Hongping Chen, Fu Wang, Yuan Hu, Youping Liu

**Affiliations:** a Department of Pharmacy, Chengdu University of Traditional Chinese Medicine, Chengdu 611137, China; b State Key Laboratory of Southwestern Chinese Medicine Resources, Chengdu 611137, China

**Keywords:** Zhoupigan, Volatile metabolites, Widely targeted metabolomics, Antioxidant, Hypolipidemic, Hypoglycemic

## Abstract

Zhoupigan (*Citrus reticulata* cv. Manau Gan) is a local citrus variety in China. Its peel, known as Zangju peel (ZJP). The metabolic profile and bioactivity of ZJP have not been adequately studied, resulting in underutilization of ZJP and a serious waste of resources. In this study, GC–MS identified 46 components in ZJP, which defined ZJP's distinct aroma. Furthermore, UPLC-ESI-MS/MS detected 1506 metabolites in ZJP, and the differential metabolites were primarily involved in the biosynthesis of flavonoids and phenylacetone. Additionally, 56 key differential metabolites with metabolic pathways were identified. ZJP had significant antioxidant activity and the enzyme inhibitory activity ranking as pancreatic lipase (IC_50_ = 3.71 mg/mL) > α-glucosidase (IC_50_ = 6.28 mg/mL) > α-amylase (IC_50_ = 8.02 mg/mL). This study aimed to evaluate the potential of ZJP as natural antioxidant and functional food source and to serve as foundation for the further development of ZJP products with specific functional attributes.

## Highlights

• The metabolic profile of Zhoupigan (*Citrus reticulata* cv. Manau Gan) was resolved.
• A total of 1535 metabolites were identified, more than previously reported.
• Metabolite profiles and bioactivities of different citrus peels were compared.
• Zhoupigan peel has excellent antioxidant and enzyme-inhibitory activities.
• Zhoupigan peel is a potential source of natural antioxidants and a functional food.

## Graphical abstract

**Figure:**
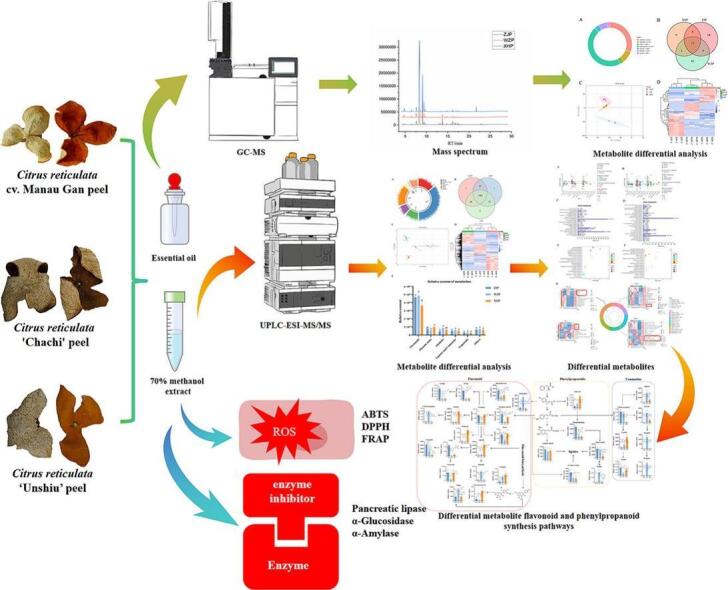

## 1 Introduction

Citrus is among the most consumed and extensively distributed fruits globally, renowned for its distinctive flavor and high nutritional value (Wang, Wang, Xiao, et al., 2024). Annually, over 130 million tons of citrus are produced, predominantly for pulp consumption, juice processing, and culinary flavoring. However, the utilization of citrus peels remains limited, leading to the generation of >15 million tons of citrus waste each year (Leporini et al., 2020), which represents a significant resource waste and environmental pollutant. Citrus peels, a primary source of essential oils (EO) stored in citrus oil glands, emit a pleasant aromatic scent (Wang, Ren, Zhou, et al., 2024). Additionally, these peels are abundant in phenolic acids, flavonoids, alkaloids, limonin, and numerous other bioactive compounds that exhibit antioxidant, anti-inflammatory, and lipid-lowering properties (Liu et al., 2021). Consequently, citrus peels can serve as a source of functional ingredients and preservatives. They are often processed into health food products such as candied fruit, jiushichenpi, Ganpu tea, or utilized as food flavoring agents. The dried ripe peel of *Citrus reticulata* and its cultivated variants, known as “chenpi” (Citri reticulatae pericarpium, CP), has been used for millennia in China for its extensive bioactivities, often as herbal medicine, flavoring, and health food. Despite numerous studies on locally cultivated citrus variants (Costanzo et al., 2022; Tao et al., 2014; Zhang et al., 2014), little research has focused on the peel of Zhoupigan, hindering the optimal utilization of Zhoupigan resources.

Zhoupigan, also known as Zangju or Shitougan, is distinguished by its wrinkled rind, succulent flesh, and a distinctively acidic, slightly bitter taste, which is highly esteemed for its unique flavor (Chen et al., 2021). Cultivated predominantly in the agricultural and pastoral regions along the middle and lower reaches of the Sanjiang River Valley, particularly in Derong and Muli counties (Sichuan Province, China), Zhoupigan covers over 11,600 ha with an annual yield of approximately 130,000 tons (https://www.nongjixie.org/Library/Nmap/view?nmap_id=1516). It is also cultivated in Ankang (Shanxi Province, China) and Yueyang (Hunan Province, China). In the Tibetan region, Zhoupigan is referred to as “Jia Xu,” meaning “longevity of the fruit.” Unlike ordinary citrus trees that typically survive for 40 to 50 years, Zhoupigan trees can flourish for nearly a century, remaining verdant and` robust. The Tibetan area is characterized by high altitude, dry climate with little rain, abundant light, and large temperature differences between day and night. As a result of this unique environment and location, Tibetans have developed distinctive dietary habits. The Tibetan diet primarily consists of high-calorie foods such as Zanba (a flour made from hulless barley), Tibetan milk tea (made from milk and sugar), and buttered tea (a drink made from salt, tea, and milk-derived oil) (Wang, Wang, Shi, et al., 2023; Xiao et al., 2021). However, the consumption of fruits and vegetables is inadequate. In this case, as a fruit cultivated on a large scale in Tibetan areas, Zhoupigan plays an important role in protecting the health of Tibetans. Local residents also incorporate ZJP into meat stews to reduce greasiness and immerse ZJP in water to relieve headaches and coughs. Due to the lack of vegetables and fruits in Tibetan areas, Tibetans generally store Zhoupigan after harvesting for approximately two months to prolong consumption time. In addition, Tibetans believe that storage helps to increase the flavor of Zhoupigan. Therefore, the limited research on Zhoupigan, a widely cultivated citrus variety with a distinct flavor and diverse uses grown in a unique environment, has obstructed its full utilization. It further emphasizes the significance of conducting a comprehensive assessment of its metabolite profile and bioactivity. In our previous research, utilizing HS-GC-IMS analyzed the volatile components of ZJP at different maturity stages, identifying some components not reported in other citrus varieties (Wang, Wang, Zou, et al., 2023).

Hyperglycemia and hyperlipidemia commonly coexist in patients with diabetes (Wang, Ren, et al., 2022). Pancreatic lipase (PL) is essential for the hydrolysis of dietary triglycerides, and lipase inhibitors can produce a hypolipidemic effect, aiding in the management of metabolic disorders (Zeng et al., 2018). α-amylase and α-glucosidase are essential enzymes in starch digestion. α-amylase catalyzes the hydrolysis of starch by breaking the α-1,4-glucosidic bond, followed by α-glucosidase further degrading hydrolysis products into glucose, thus increasing glucose levels. Inhibiting these enzymes can help control blood glucose levels (Wang, Hu, Li, et al., 2024). Currently, Orlistat (ORL) is the only approved PL inhibitor, while hypoglycemic drugs include acarbose (ACA), miglitol, and voglibose; however, these enzyme inhibitors often have side effects (Wu et al., 2013; Zhang et al., 2017). Natural plants have long been a source of medicines; phytotherapy, which uses plant-derived medicines, is often safer and more effective with fewer side effects than chemical medicines. Plant by-products are an important source of phenolic compounds. Phenol extraction from by-products (stems, leaves, and stem-leaf mixtures) of safflower (*Carthamus tinctorius* L.) and analysis of their composition by UPLC-DAD-MS, followed by assessment of their antioxidant and erythrocyte protective properties, revealed that that they are an ideal source of phenolics that can be used in the food and pharmaceutical sectors (Del-Toro-Sánchez et al., 2021). Plants such as *Allium cepa* L. and *Panax ginseng* C. A. Mey. have been utilized in diabetes treatment (Governa et al., 2018). Citrus peels, rich in various bioactive components, are an ideal source of dietary supplements. Functional foods prepared by incorporating citrus extracts or utilizing citrus by-products were shown to exhibit more potent antioxidant capacity (Gómez-Mejía et al., 2023; Laganà et al., 2022; Peng et al., 2022). Research by Huang, Zhang, et al. (2020) identified hesperidin as the primary PL inhibitor, while Huang, Zhu, et al. (2020) suggested that the PL inhibitory activity of citrus peels might be due to their polymethoxyflavonoids (PMFs) content. Phenols extracted from citrus peels exhibit strong antioxidant, antidiabetic, and antihypertensive properties (Alu'datt et al., 2017; Qurtam et al., 2021), and citrus EOs significantly inhibit α-Glucosidase and α-Amylase, demonstrating potential hypoglycemic and antidiabetic effects (Benayad et al., 2021). Currently, studies on the *in vitro* antidiabetic activity of citrus extracts have mainly focused on either hypolipidemic or hypoglycemic properties individually, while reports on their combined properties are rare and mostly evaluate the activity of a certain class of compounds, such as flavonoids, phenols, and PMFs (Alu'datt et al., 2017; Kong et al., 2020; Loizzo et al., 2019; Zeng et al., 2018). This can hinder our ability to thoroughly assess their hypolipidemic and hypoglycemic activities. However, while the inhibitory activity of citrus extracts on pancreatic lipase, α-glucosidase, and α-amylase has been confirmed by numerous studies, it has not been adequately demonstrated how these extracts or individual compounds inhibit these enzymes. We previously evaluated ZJP's antioxidant and hypolipidemic activities across different maturation stages and revealed excellent antioxidant capacity, lipase inhibition, the ability to inhibit lipid differentiation of 3 T3-L1 cells, and promotion of lipid metabolism. (Wang, Wang, Xiao, et al., 2024). In conclusion, citrus peels exhibit strong hypolipidemic and hypoglycemic activities, and while ZJP also shows excellent antioxidant and hypolipidemic activities, its hypoglycemic potential remains unexplored, and comparisons with other citrus peels' bioactivities are yet to be reported.

Metabolomics is an effective technology for detecting compounds, yet the diversity of secondary metabolites in plants presents significant identification challenges, thereby impeding plant metabolome detection. UPLC-ESI-MS/MS-based widely targeted metabolomics is an emerging analytical technology, combining the “versatility” of untargeted metabolomics with the “accuracy” of targeted metabolomics. Widely targeted metabolomics utilizes QTRAP mass spectrometry in multiple reaction monitoring (MRM) mode, enabling the qualitative and quantitative detection of over 1800 primary and 28,000 secondary metabolites in a single run. This method has gained widespread application in food and drug research due to its high sensitivity, specificity, and throughput (Jia et al., 2023; Qian et al., 2023; Yang et al., 2023; Zheng et al., 2023). Recently, widely targeted metabolomics has been extensively employed to study specificities among different varieties, sites, maturity stages, and colors (Dadwal et al., 2022; Li, Wen, et al., 2021; Shen et al., 2023; Wang, Wang, Xiao, et al., 2024). Consequently, comparing ZJP with other citrus peel metabolites using widely targeted metabolomics can elucidate the similarities and differences among the metabolites more effectively.

Although we have previously conducted preliminary studies on ZJP composition and activity, we have not analyzed the composition of ZJP's EO or compared its composition and activity with other citrus peels, which limits the use of ZJP. Therefore, GC–MS and UPLC-ESI-MS/MS-based widely targeted metabolomics were initially implemented to analyze the composition of ZJP. Its composition was then compared with that of *C. reticulata* ‘Unshiu’ peel (WZP) and *C. reticulata* ‘Chachi’ peel (XHP), which are included in the Chinese Pharmacopoeia, to assess their metabolite profile differences. Subsequently, multivariate analyses such as principal component analysis (PCA) and partial least squares discriminant analysis (OPLS-DA) were conducted to extract valuable insights from the metabolomics data. In addition, differences in PL, α-glucosidase, α-amylase inhibitory activity and antioxidant activity between ZJP and XHP and WZP were compared, and these differences were correlated with key differential metabolites (DMs). In addition, we compared the hypolipidemic and hypoglycemic-related enzyme inhibitory activities in the peels from the different citrus genotypes. This study aimed to assess ZJP's potential and feasibility as a natural antioxidant and functional food source and to compare the metabolic profiles and *in vitro* bioactivities between ZJP, XHP and WZP.

## 2 Materials and methods

### 2.1 Materials and reagents

#### 2.1.1 Plant material

Zhoupigan was sourced from Derong County, Ganzi Tibetan Autonomous Region, Sichuan Province, China (99°16′37′′ E; 28°32′32′′ N; 2225 m). *C. reticulata* ‘Unshiu’ was obtained from Danling County, Meishan City, Sichuan Province, China (103°30′57.3″ E; 30°0′54.0″ N; 509 m). Upon collection, the samples were transported to the laboratory, where the peels were manually washed, separated, and dried using hot air at a constant temperature of 50 °C (101–3 A, Beijing Zhongxingweiye Century Instrument Co., Ltd., Beijing, China) for 18 h to produce the Zhoupigan peel (ZJP) and *C. reticulata* ‘Unshiu’ peel (WZP). These peels were then stored in a dry place at room temperature (25–28 °C). *C. reticulata* ‘Chachi’ peel (XHP) was procured in 2023 from Xinhui District, Jiangmen County, Guangdong Province, China (113°01′52″ E; 22°31′41″ N) as a one-year storage sample. The samples are illustrated in Fig. S1.

#### 2.1.2 Chemicals

Methanol, acetonitrile, and formic acid were purchased from Merck Drugs & Biotechnology, USA, and were chromatographically pure. 1,1-diphenyl-2-trinitrophenylhydrazine (DPPH), 2,2′-azino-bis-3-ethylbenzothiazoline-6-sulfonic acid (ABTS), 4-nitrophenyl laurate, *p*-nitrophenyl-α-D-glucopyranoside (PNPG), and α-glucosidase (α-glu) were purchased from Shanghai Macklin Biochemical Technology Co., Ltd., (Shanghai, China). Total antioxidant capacity (T-AOC) assay kit (ferric ion reducing antioxidant capacity, FRAP) was purchased from Nanjing Jiancheng Biotechnology Research Institute Co., Ltd., (Nanjing, Jiangsu Province, China). α-amylase was purchased from Sigma Aldrich (Shanghai) Trading Co., Ltd., (Shanghai, China). Porcine PL was purchased from Shanghai yuanye Bio-Technology Co., Ltd., (Shanghai, China). Soluble starch was provided by China National Institute for the Control of Pharmaceutical and Biological Products (Beijing, China). All the above reagents were analytically pure.

### 2.2 Sample preparation and extraction for GC–MS

For essential oil extraction, 100 g of dried sample powder was soaked in 10 times the volume of water for 2 h. The essential oil was then extracted using a volatile oil extractor until no further increase in oil quantity was observed. The upper essential oil was carefully collected and dried with about 0.5 g of anhydrous sodium sulfate at 4 °C for 24 h. The essential oil was diluted 100-fold with n-hexane, filtered through a microporous filter membrane (pore size 0.22 μm), and prepared for GC–MS analysis.

### 2.3 GC–MS conditions

Gas chromatography-mass spectrometry (GC–MS) was conducted using an Agilent 7890 A-5975C gas chromatograph equipped with an HP-5 MS capillary column (0.25 mm × 30 m, 0.25 μm, Agilent J&W Scientific, Folsom, CA, USA). The carrier gas was high-purity He (99.999%), with an injection volume of 1 μL and a split ratio of 10:1, at a flow rate of 1.0 mL/min. The heating program started at an initial temperature of 40 °C, maintained for 3.5 min, followed by an increase of 10 °C/min to 100 °C, then 25 °C/min to 260 °C, held for 5 min. The EI ion source operated at 70 eV, with an ion source temperature of 230 °C, a quadrupole temperature of 150 °C, an interface temperature of 250 °C, a solvent delay of 3.0 min, and in full scan mass scan mode. The NIST 11 (2011), NIST 14 (2014), and RTLPEST 3 (2011) standard spectral libraries were searched, and the relative percentage content of each component was calculated using the peak area normalization method.

### 2.4 Sample preparation and extraction for widely targeted metabolomic analysis

Using a vacuum freeze-drying technique, samples were placed in a lyophilizer (Scientz-100F, Ningbo Xinzhi Bio-technology Co., Ltd., Ningbo, Zhejiang Province, China) and subsequently ground into a fine powder using a grinder (MM 400, Verder Shanghai Instruments and Equipment Co., Ltd., Shanghai, China) at 30 Hz for 1.5 min. Precisely 50 mg of the powdered sample was weighed using an electronic balance (MS105DΜ, Mettler Toledo Group, Zurich, Switzerland) and combined with 1200 μL of −20 °C pre-cooled 70% methanol aqueous solution. The mixture was vortexed every 30 min for 30 s, repeated six times. After centrifugation at 12,000 rpm for 3 min, the supernatant was aspirated, filtered through a microporous filter membrane (pore size 0.22 μm), and stored in an injection vial for UPLC-MS/MS analysis.

### 2.5 UPLC–MS/MS conditions

The sample extracts were analyzed using an UPLC-ESI-MS/MS system (UPLC, ExionLC™ ADˈ https: //sciex.com.cn/; MS, Applied Biosystems 6500 Q TRAP, https://sciex.com.cn/). The analytical conditions were as follows, UPLC: column, Agilent SB-C_18_ (1.8 μm, 2.1 mm * 100 mm); The mobile phase was consisted of solvent A, pure water with 0.1% formic acid, and solvent B, acetonitrile with 0.1% formic acid. Sample measurements were performed with a gradient program that employed the starting conditions of 95% A, 5% B. Within 9 min, a linear gradient to 5% A, 95% B was programmed, and a composition of 5% A, 95% B was kept for 1 min. Subsequently, a composition of 95% A, 5.0% B was adjusted within 1.1 min and kept for 2.9 min. The flow velocity was set as 0.35 mL per minute; The column oven was set to 40 °C; The injection volume was 2 μL. The effluent was alternatively connected to an ESI-triple quadrupole-linear ion trap (QTRAP)-MS.

The ESI source operation parameters were as follows: source temperature 500 °C; ion spray voltage (IS) 5500 V (positive ion mode)/−4500 V (negative ion mode); ion source gas I (GSI), gas II(GSII), curtain gas (CUR) were set at 50, 60, and 25 psi, respectively; the collision-activated dissociation(CAD) was high. QQQ scans were acquired as MRM experiments with collision gas (nitrogen) set to medium. DP(declustering potential) and CE(collision energy) for individual MRM transitions was done with further DP and CE optimization. A specific set of MRM transitions were monitored for each period according to the metabolites eluted within this period.

### 2.6 Identification and quantification of metabolites

Mass spectrometry data were processed using Analyst 1.6.3 software for qualitative and quantitative analysis of metabolites. Total ion chromatography (TIC) illustrated chromatographic differences (Fig. S2), while the MRM Metabolite Assay Multi-Peak Chart depicted detectable metabolites, with each color representing a distinct metabolite (Fig. S3). Qualitative analysis was conducted by comparing accurate precursor ion (Q1) and product ion (Q3) values and retention times (RT), matching these with the self-built database MWDB (MetWare Biological Co., Ltd., Wuhan, Hebei Province, China). Quantitative analysis involved MRM analysis using a mass spectrometer to acquire metabolite data from different samples. Integration and calibration of chromatographic peaks were performed using MultiQuant (AB SCIEX, Framingham, MA, USA) software to calculate the relative concentration of each substance in the peak area (Fig. S4).

### 2.7 Biological activity evaluation

#### 2.7.1 Sample extraction

For further analysis, 0.2 g of ground sample powder was mixed with 10 mL of 70% methanol and extracted by ultrasonication (SB25-12D, Ningbo Xinyi Ultrasonic Equipment Co., Ltd., Ningbo, Zhejiang Province, China) for 1 h at 100 W and 50 Hz. The mixture was then centrifuged at 4000 rpm for 20 min using a centrifuge (TDZS-WS, Hunan Xiangyi Laboratory Instrument Development Co., Ltd., Changsha, Hunan Province, China). The supernatant was collected, filtered through a microporous membrane, diluted as needed, and subjected to antioxidant activity, PL, α-glucosidase, and α-amylase inhibitory activities within 2 weeks.

#### 2.7.2 Evaluation of antioxidant activity

The DPPH and ABTS free radical scavenging capacities, along with the FRAP total antioxidant capacity, of various concentrations of citrus peel extracts (0.1–5 mg/mL) were assessed following the methodology of Wang, Wang, Xiao, et al. (2024). The DPPH and ABTS free radical scavenging capacities were quantified as the semi-inhibitory concentration (IC_50_), while the total antioxidant capacity was represented in milligrams of FeSO_4_ per gram of dry weight sample (mg FeSO_4_ g^−1^ DW). The standard curve was defined as y = 3.1387× + 0.0217 (R^2^ = 0.9999).

#### 2.7.3 Evaluation of inhibition capacity of enzyme activity

The PL inhibitory capacity of citrus peel extracts (1–20 mg/mL) was measured following the method outlined by Wang, Wang, Xiao, et al. (2024), with ORL serving as a positive control. Similarly, the α-glucosidase and α-amylase inhibitory capacities of the citrus peel extracts (1–20 mg/mL) were determined using the method described by Wang, Li, et al. (2022), with ACA as a positive control. Results were reported as IC_50_ values.

### 2.8 Data analysis

PCA, Venn diagrams, Metabolite Classification Charts, and Correlation analyses were conducted using the Metware Cloud, a complimentary online platform for data analysis (https://cloud.metware.cn). The pheatmap package in R software was utilized for clustering heatmap plotting, while the MetaboAnalystR package in R software was employed to compute VIP values, rankings, and rating plots in OPLS-DA. DMs were screened using the criteria of FC ≥ 2 or FC ≤ 0.5 and VIP > 1. Statistical analyses, including bar graphs, line plots, and ANOVA, were performed using GraphPad Prism 8.4.3 (GraphPad Software, USA), with a *P*-value of <0.05 indicating statistical significance. Metabolites were annotated through the KEGG database (compounds: https://www.kegg.jp/kegg/compound/;Pathways,http://www.kegg.jp/kegg/pathway.html).

## 3 Results and discussion

### 3.1 GC–MS analysis of ZJP

**Fig. 1:**
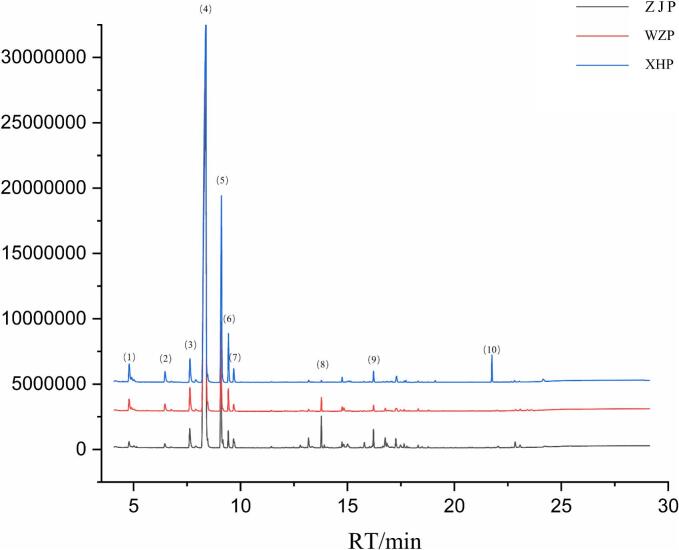
GC chromatograms of the VOCs of ZJP, WZP, and XHP. (1) L-(−)-alpha-pinene; (2) β-Pinene; (3) β-Myrcene; (4) d-Limonene; (5) γ-Terpinene; (6) o-Cymene; (7) Terpinolene; (8) Linalool; (9) α-Terpineol; (10) methyl *n*-methylanthranilate.

Citrus peel is a major source of EOs, containing numerous volatile organic compounds (VOCs) that determine the unique aroma and flavor of citrus. In this study, the VOCs of ZJP EO were analyzed *via* GC–MS and compared with those of WZP EO and XHP EO. A total of 67 VOCs, including 32 hydrocarbons, 20 alcohols, 5 aldehydes, 4 phenols, 2 esters, 2 ketones, 1 ether, and 1 pyran, were identified across the three EOs (Table 1 and Fig. S5A). PCA revealed significant separation among the three citrus peels, with ZJP distinctly diverging from WZP and XHP, which was further corroborated by clustered heatmaps (Fig. S5C-D). d-Limonene, γ-Terpinene, β-Myrcene, and linalool were the predominant compounds found in all three citrus peel EOs. Notably, ZJP contained 46 VOCs, 18 of which, such as (+)-2-Carene, Caryophyllene, and carvacrol, were unique to ZJP and exhibited woody, herbal, and waxy odors (Fig. 1, Fig. S5B). To explore the differences between ZJP and the other citrus peel VOCs, differentially abundant VOCs were identified using criteria of FC ≥ 2 or FC ≤ 0.5 and VIP > 1. A total of 28 differential VOCs were identified between ZJP and WZP, with 18 significantly upregulated in ZJP (*P* < 0.05); similarly, 28 differential VOCs were found between ZJP and XHP, with 22 significantly upregulated in ZJP (P < 0.05). Decanal, 2,6-Octadiene, 2,6-dimethyl-, (+)-Δ-Cadinene, dl-Perillaldehyde, Carveol, Carvone, (*R*)-(+)-citronellal, and Nerol were consistently upregulated in ZJP in both comparisons. These eight components, along with the 18 unique to ZJP, contribute to its distinctive flavor profile. Liu et al. (2024) characterized the flavor metabolic profiles of XHP tea liquor with varying aging times and identified d-limonene, α-phellandrene, γ-terpinene, and dimethyl anthranilate as characteristic aroma compounds of XHP.

**Table 1:** GC–MS analysis of volatile components in the essential oil of ZJP, WZP, and XHP.

No.	Compounds	RT(min)	CAS	Formula	Molecular weight	Major ions	Relative amount (%)			Class
							WZP	ZJP	XHP	
1	L-(−)-α-pinene	4.801	7785-26-4	C10H16O	152.233	93, 91, 92, 77	1.24 ± 0.01	0.58 ± 0.01	1.81 ± 0.03	Hydrocarbons
2	α-thujene	4.911	2867-05-2	C10H16	136.234	93, 91, 77, 92	0.24 ± 0.21	ND	0.51 ± 0.03	Hydrocarbons
3	Camphene	5.644	79–92-5	C10H16	136.234	93, 121, 79, 91	0.06 ± 0.01	ND	ND	Hydrocarbons
4	β-Pinene	6.474	127–91-3	C10H16	136.234	93, 69, 91, 41	0.77 ± 0.01	0.42 ± 0.00	1.13 ± 0.02	Hydrocarbons
5	β-Phellandrene	6.761	555–10-2	C10H16	136.234	93, 91, 77, 79	0.16 ± 0.01	0.10 ± 0.01	0.09 ± 0.01	Hydrocarbons
6	β-Myrcene	7.641	123–35-3	C10H16	136.234	93, 69, 41, 91	2.60 ± 0.17	1.87 ± 0.04	2.37 ± 0.08	Hydrocarbons
7	α-terpinene	7.909	99–86-5	C10H16	136.234	121, 93, 136, 91, 77	0.28 ± 0.02	ND	ND	Hydrocarbons
8	4-Carene	7.909	29,050–33-7	C10H16	136.234	121, 93, 136, 91, 76	ND	0.27 ± 0.02	0.38 ± 0.03	Hydrocarbons
9	d-Limonene	8.379	5989-27-5	C10H16	136.234	93, 68, 67, 79	77.34 ± 1.34	70.92 ± 1.30	66.79 ± 0.68	Hydrocarbons
10	γ-Terpinene	9.088	99–85-4	C10H16	136.234	93, 91, 136, 77	7.25 ± 0.32	9.61 ± 0.45	15.09 ± 0.31	Hydrocarbons
11	o-Cymene	9.436	527–84-4	C10H14	134.218	119, 134, 91, 117	1.54 ± 0.08	1.04 ± 0.07	3.01 ± 0.03	Hydrocarbons
12	Terpinolene	9.68	586–62-9	C10H16	136.234	121, 93, 136, 91, 79	0.71 ± 0.06	0.91 ± 0.08	1.00 ± 0.06	Hydrocarbons
13	3-Methyl-2-buten-1-ol	10.016	556–82-1	C5H10O	86.132	71, 41, 43, 53	ND	ND	0.02 ± 0.03	Alcohols
14	p-1,3,8-menthatriene	11.444	18,368–95-1	C10H14	134.218	57, 41, 119, 91	ND	0.04 ± 0.06	ND	Hydrocarbons
15	p-Mentha-1,5,8-triene	11.963	21,195–59-5	C10H14	134.218	91, 134, 119, 117	ND	0.01 ± 0.01	ND	Hydrocarbons
16	2-p-Tolyl-1-propene	12.098	1195-32-0	C10H12	132.202	132, 117, 115, 91	0.05 ± 0.01	0.04 ± 0.01	0.01 ± 0.01	Hydrocarbons
17	1,2-diethylbenzene	12.177	135–01-3	C10H14	134.218	91, 119, 134, 105	ND	0.01 ± 0.02	ND	Hydrocarbons
18	trans-limonene oxide	12.47	4959-35-7	C10H16O	152.233	43, 67, 94, 108	ND	0.07 ± 0.02	ND	pyrans
19	(*R*)-(+)-citronellal	12.812	2385-77-5	C10H18O	154.249	69, 41, 95, 55	0.08 ± 0.01	0.24 ± 0.02	ND	aldehydes
20	(+)-2-Carene	12.91	4497-92-1	C10H16	136.234	121, 93, 136, 161, 91	ND	0.09 ± 0.01	ND	Hydrocarbons
21	Δ-elemene	12.916	20,307–84-0	C15H24	204.35	121, 93, 136, 91, 161	0.09 ± 0.01	ND	ND	Hydrocarbons
22	Decanal	13.185	112–31-2	C10H20O	156.265	57, 41, 43, 55	0.16 ± 0.02	0.66 ± 0.06	0.14 ± 0.02	aldehydes
23	α-Cubebene	13.356	17,699–14-8	C15H24	204.351	119, 105, 161, 93	ND	0.20 ± 0.02	ND	Hydrocarbons
24	Linalool	13.783	78–70-6	C10H18O	154.249	71, 93, 43, 55	0.79 ± 0.03	1.43 ± 0.11	0.10 ± 0.00	Alcohols
25	1-Octanol	13.923	111–87-5	C8H18O	130.228	56, 55, 41, 69	ND	0.15 ± 0.01	ND	Alcohols
26	Germacrene D	14.039	23,986–74-5	C15H24	204.35	161, 105, 91, 119	ND	0.07 ± 0.07	ND	Hydrocarbons
27	β-ylangene	14.638	20,479–06-5	C15H24	204.35	161, 120, 91, 149	ND	0.09 ± 0.01	ND	Hydrocarbons
28	2-Isopropyl-5-methylanisole	14.644	1076-56-8	C11H16O	164.244	149, 164, 91, 117	ND	ND	0.02 ± 0.02	Phenols
29	4-terpineol	14.748	562–74-3	C10H18O	154.249	71, 111, 93, 43	0.29 ± 0.02	0.33 ± 0.03	0.29 ± 0.03	Alcohols
30	β-elemene	14.833	515–13-9	C15H24	204.351	81, 93, 67, 107	0.40 ± 0.03	0.33 ± 0.03	ND	Hydrocarbons
31	Caryophyllene	15.01	87–44-5	C15H24	204.351	93, 133, 91, 79	ND	0.55 ± 0.08	ND	Hydrocarbons
32	isocaryophyllene	15.016	118–65-0	C15H24	204.351	93, 91, 133, 69	ND	ND	0.09 ± 0.08	Hydrocarbons
33	β-terpineol	15.163	138–87-4	C10H18O	154.249	71, 93, 43, 107	ND	ND	0.08 ± 0.01	Alcohols
34	γ-Elemene	15.597	29,873–99-2	C15H24	204.351	121, 93, 107, 91	0.06 ± 0.01	ND	ND	Hydrocarbons
35	(*E*)-p-Menth-2,8-dien-1-ol;	15.756	7212-40-0	C10H16O	152.233	109, 134, 79, 137	ND	ND	0.05 ± 0.04	Alcohols
36	2,6-Octadiene, 2,6-dimethyl-	15.798	2792-39-4	C10H18	138.25	81, 69, 43, 95	0.07 ± 0.01	0.38 ± 0.04	ND	Hydrocarbons
37	Humulene	16.152	6753-98-6	C15H24	204.351	93, 121, 80, 41	0.02 ± 0.04	ND	ND	Hydrocarbons
38	α-Terpineol	16.226	98–55-5	C10H18O	154.249	59, 93, 121,136	0.45 ± 0.06	1.13 ± 0.11	0.60 ± 0.04	Alcohols
39	Dodecanal	16.659	112–54-9	C12H24O	184.318	57, 43, 41, 82	ND	0.10 ± 0.09	0.05 ± 0.04	aldehydes
40	β-copaene	16.769	18,252–44-3	C15H24	204.351	69, 161, 93, 41	ND	0.81 ± 0.08	ND	Hydrocarbons
41	neryl propionate	16.775	105–91-9	C13H22O2	210.313	69, 93, 41, 43	0.26 ± 0.02	ND	ND	Esters
42	Carvone	16.854	99–49-0	C10H14O	150.218	82, 108, 54, 93	ND	0.47 ± 0.05	0.07 ± 0.01	ketones
43	Valencene	16.916	4630-07-3	C15H24	204.351	161, 91, 79, 119	0.18 ± 0.02	ND	ND	Hydrocarbons
44	(−)-cis-Isopiperitenol	17.08	96,555–02-1	C10H16O	152.233	84, 83, 41, 69	0.02 ± 0.03	0.08 ± 0.07	ND	Alcohols
45	Citronellol	17.27	106–22-9	C10H20O	156.265	69, 41, 67, 55	ND	0.66 ± 0.06	ND	Alcohols
46	α-Farnesene	17.288	502–61-4	C15H24	204.351	93, 69, 41, 107	0.29 ± 0.25	ND	0.37 ± 0.32	Hydrocarbons
47	(+)-Δ-Cadinene	17.496	483–76-1	C15H24	204.351	161, 119, 134, 105	0.16 ± 0.02	0.31 ± 0.03	0.05 ± 0.01	Hydrocarbons
48	dl-Perillaldehyde	17.654	2111-75-3	C10H14O	150.218	79, 68, 67, 107	0.12 ± 0.02	0.24 ± 0.02	0.08 ± 0.01	aldehydes
49	trans-p-mentha-1(7),8-dien-2-ol	17.734	21,391–84-4	C10H16O	152.233	109, 134, 91, 119	ND	ND	0.12 ± 0.01	Alcohols
50	Nerol	17.783	106–25-2	C10H18O	154.249	69, 41, 93, 68	0.01 ± 0.02	0.12 ± 0.01	ND	Alcohols
51	t-Butylbenzene	17.874	98–06-6	C10H14	134.218	119, 43, 59, 134	0.03 ± 0.03	ND	ND	Hydrocarbons
52	cis-carveol	18.314	1197-06-4	C10H16O	152.233	109, 84, 83, 55	0.16 ± 0.02	0.19 ± 0.02	0.05 ± 0.05	Alcohols
53	Geraniol	18.497	106–24-1	C10H18O	154.249	69, 43, 135, 41	0.02 ± 0.02	0.03 ± 0.06	ND	Alcohols
54	cherry propanol	18.503	1197-01-9	C10H14O	150.218	135, 43, 91, 150	ND	ND	0.02 ± 0.02	Alcohols
55	Carveol	18.772	99–48-9	C10H16O	152.233	84, 109, 134, 69	ND	0.12 ± 0.05	0.01 ± 0.02	Alcohols
56	cis-isocarveol	19.108	22,626–43-3	C10H16O	152.233	109, 55, 67, 41	ND	ND	0.07 ± 0.06	Alcohols
57	Menth-1-en-9-ol	19.846	18,479–68-0	C10H18O	154.249	45, 94, 121, 67	ND	0.03 ± 0.02	ND	Alcohols
58	perill alcohol	20.835	536–59-4	C10H16O	152.233	79, 67, 121, 91	ND	0.01 ± 0.02	ND	Alcohols
59	Nerolidol, cis-(+)	21.574	142–50-7	C15H26O	222.366	69, 93, 107, 41	ND	0.06 ± 0.01	ND	Alcohols
60	methyl *n*-methylanthranilate	21.758	85–91-6	C9H11NO2	165.189	165, 105, 104, 132	ND	ND	1.36 ± 0.01	Esters
61	α-elemol	22.002	639–99-6	C15H26O	222.366	59, 93, 161, 81	0.08 ± 0.01	ND	ND	Alcohols
62	Thymol	22.704	89–83-8	C10H14O	150.218	135, 150, 91, 115	0.05 ± 0.00	0.06 ± 0.00	0.05 ± 0.01	Phenols
63	o-Acetyl-p-cresol	22.857	1450–72-2	C9H10O2	150.174	135, 150, 106, 77	0.14 ± 0.01	ND	ND	ketones
64	carvacrol	22.985	499–75-2	C10H14O	150.218	135, 150, 91, 77	ND	0.03 ± 0.03	ND	Phenols
65	o-Isopropylanisole	22.991	2944-47-0	C10H14O	150.218	135, 150, 91, 136, 133	ND	0.01 ± 0.02	ND	Ether
66	o-cymen-5-ol	23.07	3228-02-2	C10H14O	150.218	135, 150, 91, 136, 107	ND	0.16 ± 0.14	ND	Phenols
67	α-sinensal	24.243	17,909–77-2	C15H22O	114.189	93, 45, 55, 134	ND	0.15 ± 0.27	0.46 ± 0.05	aldehydes

Note: Data are presented as mean ± standard deviation of three replicate measurements; RT indicates retention time; ND represents not detected.

### 3.2 Metabolomic analysis of ZJP

#### 3.2.1 Metabolite profiles

**Fig. 2:**
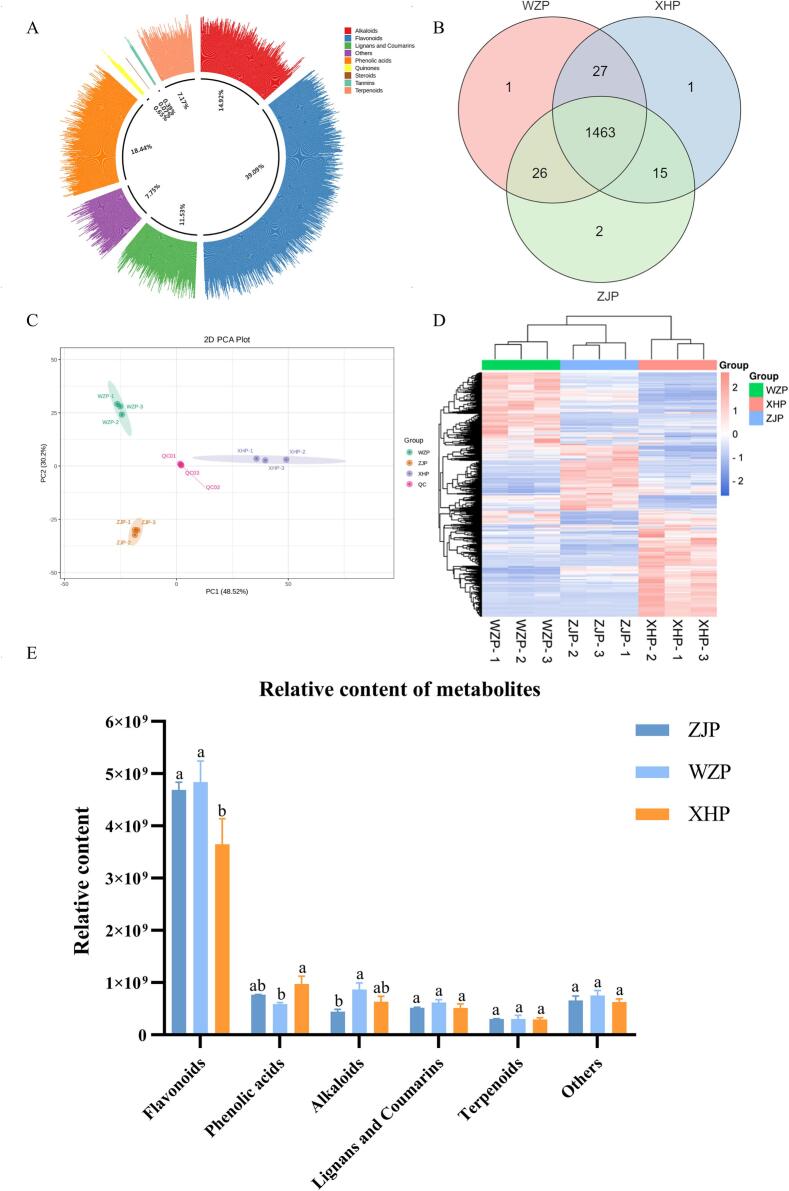
Differences and metabolite profiles between ZJP, WZP, and XHP. (A) Pie chart categorizing identified metabolites. (B) Metabolite distribution. (C) PCA analysis. (D) Heatmap of metabolite clustering. (E) Relative content of metabolites. Each peak representing a metabolite and peak height indicating relative content. Different letters in the graphs indicate significant differences in the same index (*P* < 0.05).

WZP and XHP are included in the Chinese Pharmacopoeia and are utilized as herbal medicines, benefiting from extensive and standardized cultivation bases across China. Consequently, they have been extensively studied and well-researched, unlike ZJP, which remains underexplored. To comprehensively resolve the metabolic information of ZJP and facilitate a detailed comparison of its metabolic profile with WZP and XHP, widely targeted metabolomics based on UPLC-ESI-MS/MS was employed to analyze citrus peel metabolites. A total of 1535 metabolites were detected in the three citrus peel species, classified into nine groups (Fig. 2A; Table S1): 600 flavonoids (39.09%), 283 phenolic acids (18.44%), 229 alkaloids (14.92%), 177 lignans and coumarins (11.53%), 110 terpenoids (7.17%), 10 quinones (0.65%), 6 tannins (0.39%), 1 steroid (< 0.1%), and 117 other species (7.75%). The metabolite count identified in this study surpasses that of previous studies (Wang, Chen, et al., 2022; Wang, Peng, Gao, et al., 2024; Yuan et al., 2024), highlighting the efficacy of UPLC-ESI-MS/MS-based widely targeted metabolomics in metabolite identification. The primary components of citrus peels—flavonoids, phenolic acids, and alkaloids—endow them with a broad spectrum of bioactivities, facilitating their use in functional food production. ZJP revealed 1506 metabolites, including Bergaptol and dimethoxylamine, absent in WZP and XHP (Fig. 2B). Bergaptol, a furanocoumarin commonly found in Citrus *spp.*, is non-phototoxic and non-photomutagenic. Modern pharmacological studies have demonstrated Bergaptol's anti-inflammatory, antioxidant, anticancer, anti-osteoporosis, antibacterial, and antilipidemic effects (Phucharoenrak & Trachootham, 2024), along with robust antioxidant activity and free radical scavenging abilities compared to ascorbic acid (Girennavar et al., 2007).

PCA score plot extracted two principal components (PC1 and PC2), accounting for 48.52% and 30.2% of the variance, respectively. The tight clustering of the three replicate QC samples confirmed the reproducibility and reliability of the experiment, with ZJP distinctly separated from WZP and XHP (Fig. 2C), indicating significant differences among the three citrus peels. Additionally, heatmap categorization clearly distinguished the three classes (Fig. 2D), signifying substantial differences in the metabolite content of ZJP compared to WZP and XHP. The relative contents of various metabolites are depicted in Fig. 2E, showing higher relative contents of flavonoids, alkaloids, coumarins, and lignans in WZP, while phenolic acids were more abundant in XHP. Interestingly, ZJP did not exhibit significant enrichment in a major class of compounds but displayed higher relative contents of flavones, isoflavones, flavanonols, and phenolamines compared to WZP and XHP (Fig. S6).

#### 3.2.2 Key differential metabolites

**Fig. 3:**
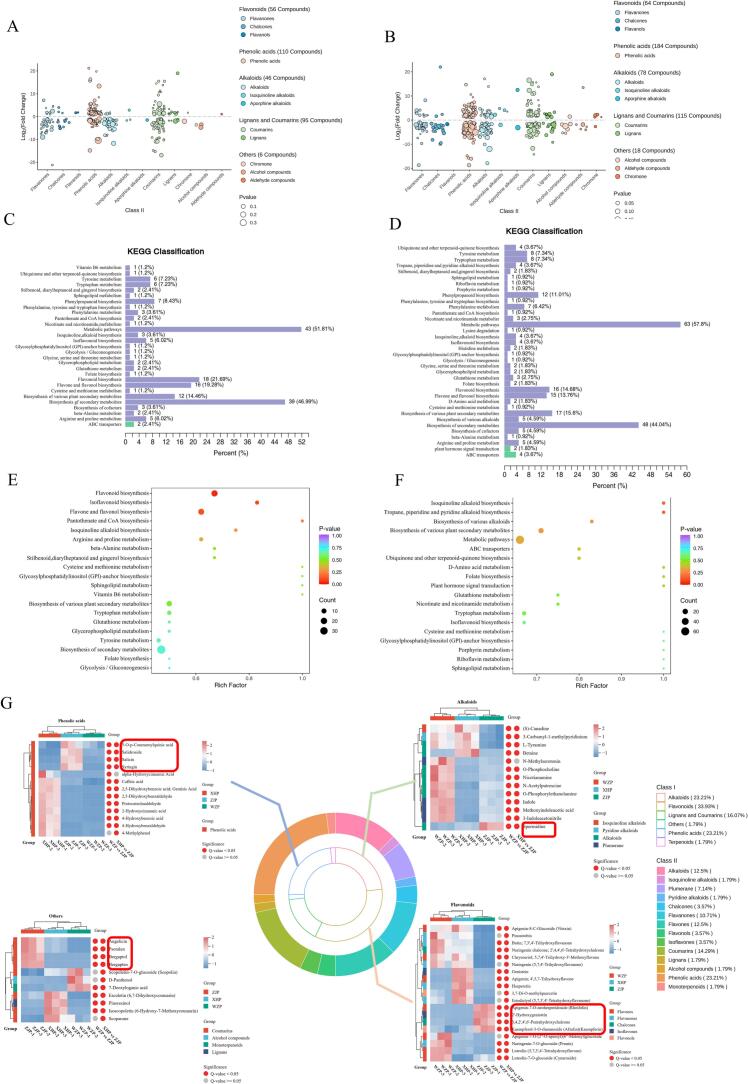
DMs analysis between ZJP *vs.* WZP and XHP. (A) WZP *vs.* ZJP DMs bubble plots. (B) XHP *vs.* ZJP DMs bubble plots. (C) WZP *vs.* ZJP KEGG pathway annotation. (D) XHP *vs.* ZJP KEGG pathway annotations. (E) WZP *vs.* ZJP KEGG enrichment analysis. (F) XHP *vs.* ZJP KEGG enrichment analysis. (G) Key DMs with clustering analysis. Bubble plots were created by selecting the top 5 Class I categories, sorted by the number of substances in Class I. For each Class I category, the top 3 Class II categories were plotted, sorted by the number of substances in Class II. The size of the points represents the *P*-value. In the KEGG enrichment plots, the horizontal coordinate indicates the Rich Factor of each pathway, the vertical coordinate lists the pathway names (sorted by P-value), the color of the dots reflects the P-value size (with red indicating more significant enrichment), and the size of the dots represents the number of enriched DMs. Metabolites circled in the clustering heatmap represent significant up-regulation in ZJP (P < 0.05). (For interpretation of the references to color in this figure legend, the reader is referred to the web version of this article.)

Comparative metabolomics comprehensively characterizes the chemical compositions across different samples, facilitating the identification of key active metabolites (Wang, Chen, et al., 2022; Wang, Hu, Chen, et al., 2023). In this study, ZJP served as the control group, with WZP and XHP as experimental groups, to analyze metabolic differences. OPLS-DA pairwise comparisons between WZP and ZJP (R^2^X = 0.778, R^2^Y = 1, Q^2^ = 0.987) and between XHP and ZJP (R^2^X = 0.801, R^2^Y = 1, Q^2^ = 0.993) assessed these differences. The Q^2^ values for all control groups exceeded 0.9, indicating model stability (Fig. S7). The distinct separation in these pairwise comparisons confirmed significant differences in their metabolic profiles (Fig. S8). DMs were screened using the criteria of FC ≥ 2 or FC ≤ 0.5 and VIP > 1, with results visualized in volcano plots (Fig. S9). Between WZP and ZJP, 731 DMs were identified, focusing on phenolic acids, lignans, coumarins, flavonoids, and alkaloids (Fig. 3A). Among these, 17 DMs, such as Psoralen and Angelicin, were unique to ZJP. Although isoflavones and phenolic acids were most abundant among the common compounds, the top five upregulated metabolites in ZJP included four lignans and coumarins—Aurapten (2706.73-fold), Bergapten (1712.67-fold), Isobergapten (1703.61-fold), and Sphondin (700.82-fold)—and the phenolic acid Protocatechuic acid glucosyl xyloside (351.56-fold). In comparisons between XHP and ZJP, 955 DMs were identified, also focusing on flavonoids, phenolic acids, lignans, coumarins, and alkaloids (Fig. 3B). Of these, 25 DMs were unique to ZJP. The top five upregulated metabolites in ZJP were Kaempferol-3-O-rutinoside-7-O-rhamnoside (2385.37-fold), Zanthoxyloside (2147.56-fold), Neoeriocitrin (1902.24-fold), Sibiricin (1814.66-fold), and Hydrangeifolin I (1598.11-fold). Additionally, PMFs were notably downregulated in ZJP, including 2’-Hydroxy-3′,4′,6′,3,4-pentamethoxychalcone (0.0010-fold), 5,6,7,8,3′,4’-Hexamethoxyflavanone (0.0012-fold), 6’-Hydroxy-2,4,2′,3′,4′,5’-Hexamethoxychalcone (0.0014-fold), and 6’-Hydroxy-4,2′,3′,4′,5’-Pentamethoxychalcone (0.0034-fold). Interestingly, while glycosides did not exhibit the most significant differences, they were consistently more upregulated in ZJP in both comparison groups.

**Fig. 4:**
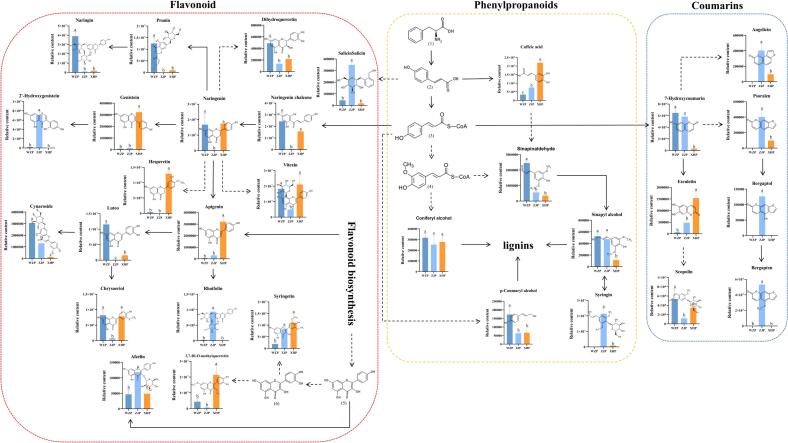
Diagram of synthetic pathways of DMs: flavonoids, lignans, and coumarins components. Different letters indicate significant differences (P < 0.05) for the same metabolite, with the absence of a letter indicating the component was not detected in the group. Solid lines indicate direct synthesis pathways, while dashed lines indicate synthesis through multiple steps. (1) L-Phenylalanine; (2) p-Coumaric acid; (3) p-Coumaroyl-CoA; (4) Feruloyl-CoA; (5) Kaempferol; (6) Quercetin.

KEGG pathway annotation revealed that most DMs were primarily involved in the biosynthesis of flavonoids, phenylpropanoids, and alkaloids, as well as amino acid biosynthesis (Fig. 3C-D). The phenylpropanoid metabolic pathway enhances plant resistance and participates in defense mechanisms through the synthesis of phenolic compounds, flavonoids, lignans, and alkaloids (Jin et al., 2009). Activation of the phenylpropanoid pathway increases the activity of related enzymes and the content of phenylpropanoids in fruits, strengthening the cell wall and preventing pathogenic bacterial invasion. This has significant implications for the preservation and antisepsis of fruits and vegetables (Wang et al., 2019). KEGG enrichment analysis demonstrated that the DMs between WZP and ZJP were mainly enriched in the biosynthesis of flavonoids, pantothenic acid, and coenzyme A (Fig. 3E). Additionally, the DMs between XHP and ZJP were involved in the biosynthesis of amino acids and alkaloids (Fig. 3F). To provide a clearer understanding of DM metabolism, the metabolic pathways of flavonoids, lignans, and coumarins were mapped based on KEGG annotation results (Fig. 4). In the flavonoid synthesis pathway, DMs were more prevalent in WZP and XHP, whereas lignans and coumarins were predominantly produced in ZJP. p-Coumaroyl-CoA is ortho-hydroxylated by cinnamoyl-CoA 2′-hydroxylase and subsequently forms 7-Hydroxycoumarin (Umbelliferone) through lactone ring closure (Phucharoenrak & Trachootham, 2024). Umbelliferone, a precursor of several coumarins, is catalyzed by marmesin synthase and psoralen synthase to form Bergaptol, a unique component of ZJP, and ultimately Bergapten, catalyzed by bergaptol 5-*O*-methyltransferase (Hehmann et al., 2004).

A Venn diagram of the DMs revealed 509 common DMs in both groups (Fig. S10), with 166 DMs significantly upregulated in both groups, indicating significant biological activity in ZJP. KEGG annotated 56 key DMs with metabolic pathways (Table S2), encompassing 19 pathways such as flavonoid biosynthesis, amino acid metabolism, and glycolysis (Fig. S11A), which were primarily enriched in secondary metabolite synthesis and phenylpropanoid biosynthesis (Fig. S11B). These included 19 flavonoids, 13 phenolic acids, 13 alkaloids, and 9 coumarins and lignans, along with 1 terpenoid and 1 other. 13 metabolites were significantly upregulated in ZJP (Fig. 3G), including Rhoifolin, Salidroside, Salicin, and Angelicin, which exhibited potent anti-inflammatory, antioxidant, and anti-osteoporotic activities.

### 3.3 Biological activity evaluation

#### 3.3.1 Antioxidant activities

**Fig. 5:**
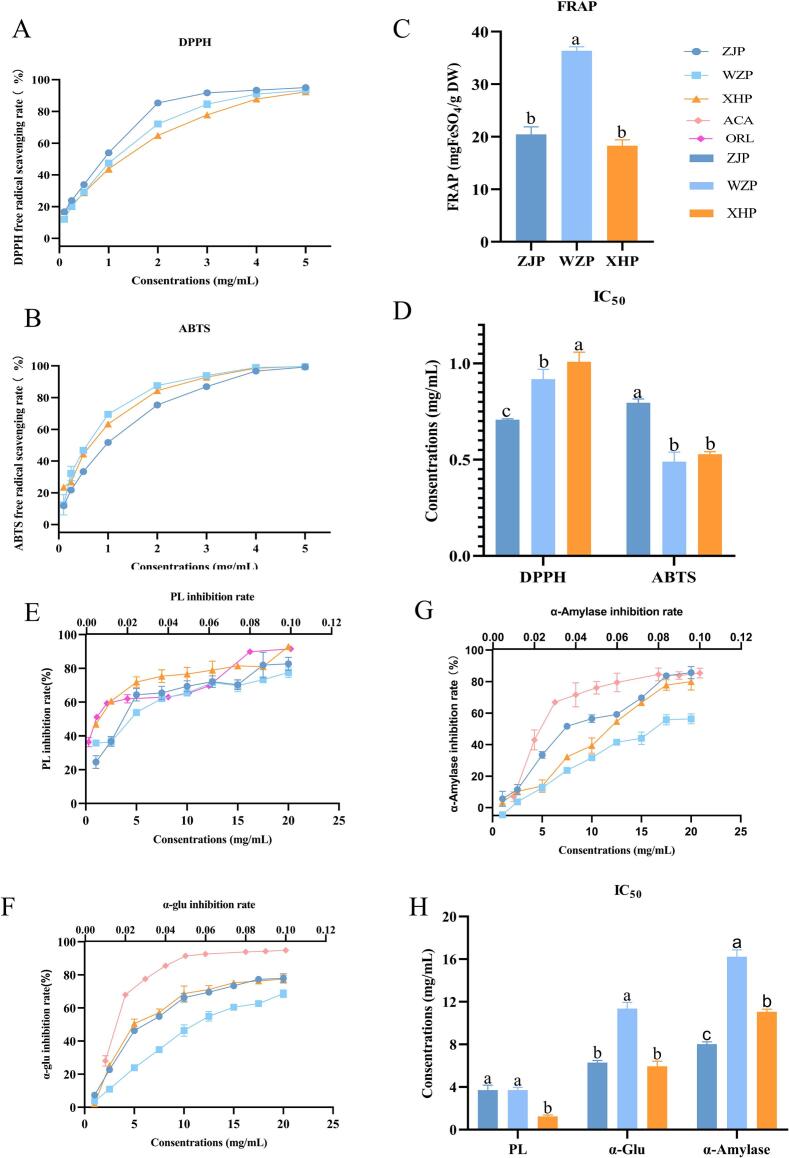
Comparison of bioactivity of three citrus peels. (A) DPPH radical scavenging capacity. (B) ABTS free radical scavenging capacity. (C) FRAP total antioxidant capacity. (D) IC_50_ value of DPPH and ABTS scavenging rates. (E) PL inhibitory activity. (F) α-glucosidase inhibitory activity. (G) α-amylase inhibitory activity. (H) IC_50_ value of enzyme activity inhibitory capacities. Different letters in the graphs indicate significant differences in the same index (P < 0.05).

Oxidative stress is linked to various chronic diseases, and natural antioxidants can effectively mitigate the damage caused by oxidative stress. Citrus peels are rich in secondary metabolites such as flavonoids and phenolic acids, which significantly contribute to human health, particularly through their antioxidant activity, making them an ideal source of natural antioxidants (Peng et al., 2022). This study evaluated the antioxidant activity of three citrus peel extracts using DPPH, ABTS, and FRAP methods. All three citrus peel extracts exhibited strong DPPH and ABTS free radical scavenging activities at various concentrations, which increased with concentration (Fig. 5A-B). The highest total antioxidant capacity, as measured by FRAP, was observed in WZP (36.33 mg FeSO_4_/g DW), significantly surpassing that of ZJP and XHP (*P* < 0.05), although no significant difference was noted between ZJP and XHP (*P* > 0.05) (Fig. 5C). The DPPH radical scavenging capacity (IC_50_) of the three citrus peel extracts ranged from 0.71 to 1.01 mg/mL, with ZJP exhibiting the strongest DPPH radical scavenging rate (IC_50_ = 0.71 mg/mL), significantly higher than WZP and XHP (P < 0.05). The ABTS radical scavenging capacity (IC_50_) ranged from 0.49 to 0.80 mg/mL (Fig. 5D), with WZP showing the strongest ABTS radical scavenging capacity (IC_50_ = 0.49 mg/mL), not significantly different from XHP (P > 0.05), but both were significantly higher than ZJP (P < 0.05). Interestingly, ZJP demonstrated the strongest DPPH radical scavenging capacity but the lowest ABTS scavenging capacity. The mechanisms of different antioxidant capacity assays vary, potentially leading to inconsistent results for the same sample across different methods (Chen et al., 2020).

The primary methods that are commonly employed for determining the *in vitro* antioxidant capacity include inhibition of lipid peroxidation, free radical scavenging capacity, ferric ion reducing capacity, and copper ion reducing capacity (Gulcin, 2020). In addition, the antioxidant capacity determination methods can be divided into those based on electron transfer and hydrogen ion transfer. However, these two mechanisms cannot distinguish between ABTS and DPPH (Apak et al., 2016). Nonetheless, the principle of the assay remains the same: measuring the scavenging capacity against colored free radicals or the ability to inhibit redox-active compounds (Floegel et al., 2011). The assessment of antioxidant capacity involves measuring changes in the color of the colored ABTS and DPPH radicals, or the colorless FRAP, following their reaction with antioxidants (Gulcin, 2020). Factors such as concentration, pH, polarity, and color of the sample can influence the results (Munteanu & Apetrei, 2021), indicating that a single model should not be used to evaluate antioxidant capacity comprehensively. Polyphenols comprise a wide range of compounds that have a benzene ring and multiple hydroxyl groups in their chemical structure. They mainly include flavonoids, phenolic acids, and other compounds. The antioxidant activity of polyphenols lies in their ability to provide hydrogen or electrons and to delocalize unpaired electrons within aromatic structures (Fernandez-Panchon et al., 2008). Polyphenol polarity has an important effect on its biological activity, with a higher number of phenolic hydroxyl groups and a higher polarity associated with a stronger antioxidant activity (Xing et al., 2020). Hagerman et al. (1998) also demonstrated that the potency of antioxidants is associated with the number of phenolic hydroxyl groups they contain. Moreover, Feng and Liu (2009) further found that their antioxidant capacity is determined by the substituted phenolic hydroxyl groups rather than enol hydroxyl groups. Interestingly, a similar discovery was made in lignans (Nsimba et al., 2012). ZJP and WZP have relatively high flavonoid content, which explains their higher antioxidant capacity. The notable antioxidant activity of citrus peels may be attributed to their rich content of phenolic acids, flavonoids, carotenoids, and other compounds (Singh et al., 2020). Specifically, the number and position of hydroxyl groups bound to the aromatic ring significantly affect the antioxidant capacity of phenolic acids (Gulcin, 2020). In conclusion, this study demonstrated that ZJP possesses substantial antioxidant activity, consistent with previous findings (Cao et al., 2023; Wang, Wang, Wang, et al., 2024; Wang, Wang, Xiao, et al., 2024), confirming it as an excellent source of natural antioxidants.

#### 3.3.2 PL inhibitory activities

PL is an essential enzyme in the lipid metabolic pathway, facilitating the conversion of accumulated lipids into fatty acids and monoacylglycerols, which are then absorbed by small intestinal epithelial cells (Li, Xue, et al., 2021). Thus, the application of natural PL inhibitors is crucial in reducing lipid accumulation and promoting lipid catabolism and absorption. This study demonstrated that all three citrus peel extracts inhibited PL activity, with the inhibitory effects intensifying as concentration increased (Fig. 5E). XHP exhibited the most potent PL inhibitory activity (IC_50_ = 1.24 mg/mL, Fig. 5H), significantly surpassing ZJP (IC_50_ = 3.71 mg/mL) and WZP (IC_50_ = 3.70 mg/mL), with no significant difference between the latter two. The robust PL inhibitory capacity of citrus peels may be attributed to the adsorption of insoluble dietary fiber to PL (Yu et al., 2023). Previous studies by Zeng et al. (2018) observed that PL inhibition in XHP progressively increased from August to December, while our earlier research noted a rise and subsequent decline in ZJP PL inhibition from October to December (Wang, Wang, Xiao, et al., 2024).

#### 3.3.3 α-glucosidase and α-amylase inhibitory activities

Hyperglycemia and hyperlipidemia often coexist in patients with diabetes, posing major risk factors for atherosclerotic cardiovascular disease. α-amylase and α-glucosidase catalyze the hydrolysis of starch into products such as maltose, maltotriose, and dextrin, leading to elevated blood glucose levels and hyperglycemia. The α-glucosidase and α-amylase inhibitory activities of ZJP, WZP, and XHP increased with concentration (Fig. 5F-G), with α-glucosidase inhibition stronger than α-amylase inhibition across all three citrus peels, though all were weaker than PL inhibition (Fig. 5H). XHP exhibited slightly stronger α-glucosidase inhibitory activity (IC_50_ = 5.92 mg/mL) compared to ZJP (IC_50_ = 6.28 mg/mL), with both significantly higher than WZP (IC_50_ = 11.35 mg/mL), reflecting an inverse trend to the FRAP total antioxidant capacity. Significant differences in α-amylase inhibitory activities were noted, with ZJP demonstrating the strongest inhibition (IC_50_ = 8.02 mg/mL), followed by XHP (IC_50_ = 11.06 mg/mL), and WZP exhibiting the weakest (IC_50_ = 16.19 mg/mL). Citrus flavonoids significantly inhibit starch digestion, with naringin and neohesperidin primarily inhibiting amylose digestion, while hesperidin and nobiletin inhibit both amylose and amylopectin digestion (Shen et al., 2012).

The dried ripe peels of *C. reticulata* ‘Chachi’ and *C. reticulata* ‘Unshiu’, known as “Chenpi” in China, are widely used as herbal medicine and food flavoring agents, and are processed into functional foods such as ‘Jiuzhichenpi’, ‘Chenpi Candy’, and ‘Ganpu Tea’. The aging process of Chenpi is a natural fermentation process (Yang et al., 2022), and “Ganpu tea” is a novel tea beverage made by co-fermenting Puer tea leaves wrapped in intact citrus peels (Deng et al., 2022). Modern pharmacological studies have demonstrated that both “Chenpi” and “Ganpu Tea” possess potent hypolipidemic and hypoglycemic activities (Huang, Zhang, et al., 2020; Wang, Gmitter Jr, et al., 2022; Zeng et al., 2018), corroborated by this study. This research further demonstrated that ZJP exhibits strong PL, α-glucosidase, and α-amylase inhibitory activities, highlighting its potential as a functional product for lowering blood lipids and glucose levels.

### 3.4 Correlation analysis

**Fig. 6:**
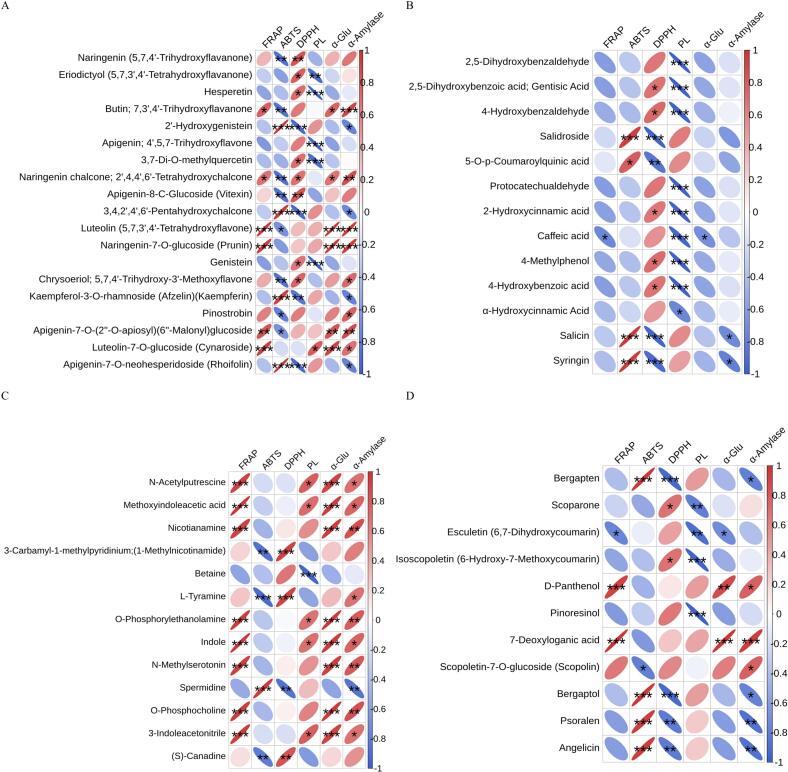
Correlation analysis of key DMs with antioxidant and enzyme activity inhibition capacity. (A) Flavonoids. (B) Phenolic acids. (C) Alkaloids. (D) Other classes. In the figure, * indicates P < 0.05; ** indicates *P* < 0.01; *** indicates *P* < 0.001.

To further analyze activity and key DM correlations, Pearson correlation analyses were conducted for ABTS, DPPH, FRAP, PL, α-glucosidase, and α-amylase inhibitory activities (all expressed as IC_50_ except FRAP), along with 56 key DMs (Fig. 6A-D). Luteolin, Luteolin-7-O-glucoside, Prunin, Nicotianamine, Indole, and 3-Indoleacetonitrile exhibited significant positive correlations with the total antioxidant capacity measured by FRAP. Conversely, L-Tyramine, Naringenin, Naringenin chalcone, Butin, Vitexin, Chrysoeriol, (*S*)-Canadine, and Scopolin showed significant negative correlations with ABTS. Metabolites such as 2’-Hydroxygenistein, 3,4,2′,4′,6’-Pentahydroxychalcone, Rhoifolin, Salidroside, Salicin, Syringin, Bergapten, Bergaptol, Angelicin, and Psoralen, which were significantly up-regulated in ZJP, demonstrated significant negative correlations with DPPH. Hesperetin, Apigenin, 3,7-Di-*O*-methylquercetin, Genistein, Protocatechualdehyde, Caffeic acid, 4-Hydroxybenzoic acid, α-Hydroxycinnamic acid, 2-Hydroxycinnamic acid, Betaine, and Pinoresinol exhibited significant negative correlations with PL inhibitory activity. Spermidine, Bergapten, Bergaptol, Psoralen, and Angelicin showed significant negative correlations with α-amylase inhibitory activity. Interestingly, only Caffeic acid and Esculetin were significantly negatively correlated with α-glucosidase inhibitory activity, while most key DMs were significantly positively correlated with α-glucosidase inhibitory activity. The correlation analysis, combined with experimental data, indicates that the activity of the samples was not solely determined by the key DMs identified. Metabolites such as Gallic acid, Rutin, Nobiletin, Quercetin, Limonin, Ferulate, Chlorogenic acid, and Naringin, although not identified as key DMs in this study, have been extensively researched for their roles in maintaining human health (Singh et al., 2020; Zhao et al., 2020). Additionally, anthocyanins and carotenoids, which were not examined in this study, also play significant roles in plant growth and human health (Rapisarda et al., 2022). In summary, this study revealed that ZJP, WZP, and XHP, along with their metabolites such as Bergapten, Hesperetin, Luteolin, Luteolin-7-O-glucoside, Naringenin, Naringenin chalcone, Chrysoeriol, 2’-Hydroxygenistein, Rhoifolin, and Cynaroside, possess potential antioxidant, hypolipidemic, and hypoglycemic activities. Furthermore, ABTS showed a significant negative correlation with DPPH, and the different mechanisms of ABTS, DPPH, and FRAP assays resulted in varying antioxidant capacities for the same sample (Chen et al., 2020). α-glucosidase and α-amylase, two key enzymes in glucose regulation, exhibited a significant positive correlation with each other and with the FRAP total antioxidant capacity.

## 4 Conclusion

This study utilized GC–MS to analyze the volatile components of ZJP, identifying 46 species, predominantly d-Limonene, γ-Terpinene, and β-Myrcene. Additionally, 18 unique components, such as Caryophyllene, Citronellol, and carvacrol, were detected exclusively in ZJP, contributing to its distinctive herbal, woody, and waxy aroma. Among the three citrus peels analyzed, a total of 1535 metabolites were detected, with 1506 found in ZJP. The DMs were concentrated in phenolic acids, lignans, coumarins, flavonoids, and alkaloids. KEGG pathway annotation indicated that most DMs were involved in the biosynthesis of flavonoids, phenylpropanoids, alkaloids, and amino acids, a finding corroborated by enrichment analysis. From the three citrus peels, 509 DMs were screened, and KEGG annotation identified 56 key DMs involved in 19 metabolic pathways, including flavonoid biosynthesis, amino acid metabolism, and glycolysis. These pathways were primarily enriched in the synthesis of secondary metabolites and phenylpropanoid biosynthesis, with 13 metabolites, such as Psoralen and Salicin, significantly up-regulated in ZJP. *In vitro* activity assays demonstrated that ZJP exhibited the strongest DPPH radical scavenging (IC_50_ = 0.71 mg/mL) and α-amylase inhibitory activity (IC_50_ = 8.02 mg/mL). WZP showed the highest ABTS radical scavenging (IC_50_ = 0.49 mg/mL) and FRAP total antioxidant capacity (36.33 mg FeSO4/g DW), while XHP had the strongest PL and α-glucosidase inhibitory activities (IC_50_ = 1.24 mg/mL and 5.92 mg/mL, respectively). The study highlighted ZJP's potential antioxidant, hypolipidemic, and hypoglycemic activities. Pearson correlation analysis revealed significant correlations between metabolites such as Bergaptol, Vitexin, and Luteolin with antioxidant activity, and Apigenin, Caffeic acid, Esculetin, Butin, and Rhoifolin with PL, α-glucosidase, and α-amylase inhibitory activities.

This study provided the first comprehensive metabolite profile of ZJP and evaluated its antioxidant, lipid-lowering, and blood sugar-lowering capacities compared to WZP and XHP. The results showed that ZJP was generally similar to WZP and XHP in terms of metabolite composition but differed in their content, with key differential metabolites annotated to pathways such as glycolysis. Meanwhile, the *in vitro* activity study found that ZJP had greater hypoglycemia capacity compared to hypolipidemic capacity. However, there is still uncertainty regarding the absorption and transformation of metabolites and the mechanism of action of ZJP in glycolipid metabolism, and we will further study the mechanisms underlying the hypolipidemic and hypoglycemia effects of ZJP through *in vivo* experiments in the future. Besides, we did not compare the composition and activity of ZJP to more citrus peels. In conclusion, our study illustrated that ZJP is an ideal source of natural antioxidants and functional ingredients. Subsequent research should be conducted to develop ZJP into functional health-promoting food products to fully utilize Zhoupigan resources.

## CRediT authorship contribution statement

**Jialiang Zou:** Writing – original draft, Visualization, Methodology, Investigation, Formal analysis, Conceptualization. **Peng Wang:** Writing – review & editing, Data curation. **Huanhuan Xu:** Writing – review & editing, Methodology, Data curation. **Xuelian Gan:** Writing – review & editing, Methodology, Data curation. **Huangsheng Zhang:** Writing – review & editing, Methodology, Data curation. **Lin Chen:** Writing – review & editing, Methodology, Data curation. **Hongping Chen:** Writing – review & editing, Methodology, Data curation. **Fu Wang:** Writing – review & editing, Methodology, Data curation. **Yuan Hu:** Validation, Formal analysis. **Youping Liu:** Writing – review & editing, Supervision, Funding acquisition, Conceptualization.

## Declaration of competing interest

The authors declare that they have no known competing financial interests or personal relationships that could have appeared to influence the work reported in this paper.

## Data Availability

Data will be made available on request.

## Appendix A Supplementary data

Supplementary material 1 Image 1

Supplementary material 2 Image 2

Supplementary material 3 Image 3
