# Supplementary material for: Metabolic profile and bioactivity of the peel of Zhoupigan (Citrus reticulata cv. Manau Gan), a special citrus variety in China, based on GC–MS, UPLC-ESI-MS/MS analysis, and in vitro assay
Source: Food Chem X. 2024 Aug 6;23:101719. doi: 10.1016/j.fochx.2024.101719 (PMC11367054; doi:10.1016/j.fochx.2024.101719)
Supplement: Supplementary file 1 — Supplementary material 1 [file mmc1.docx]

**Supplementary material**

**Fig. S1**. Plots of the 3 citrus peel samples, from left to right, ZJP, XHP, and WZP, respectively.


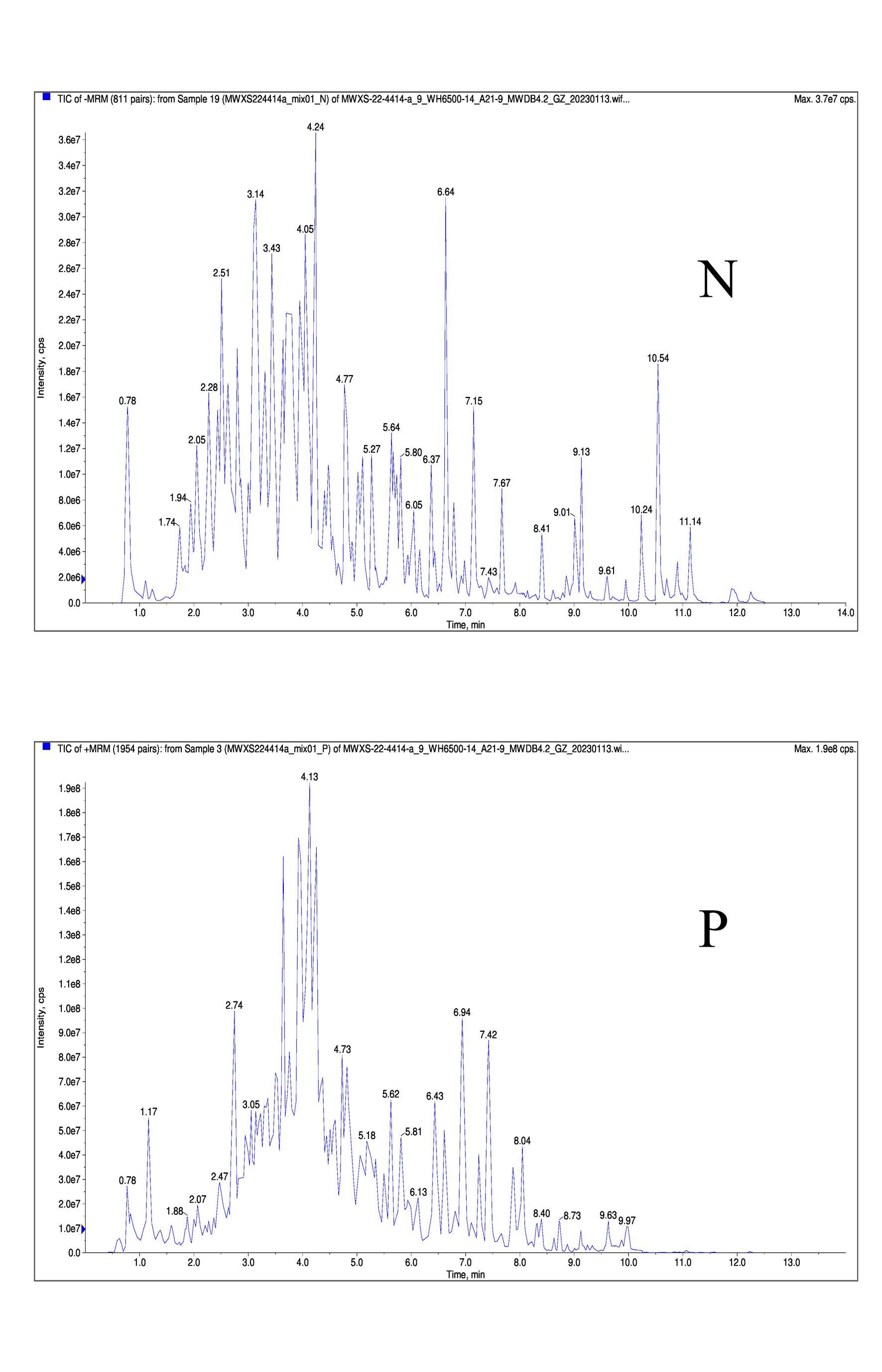


**Fig. S2.** Total ions current (TIC) overlapping map of QC samples results. Note: The abscissa is the retention time of the metabolite. The ordinate is the ion current intensity of the ion detection (the intensity units are counts per second (cps)). N stands for negative ion mode; P stands for positive ion mode.


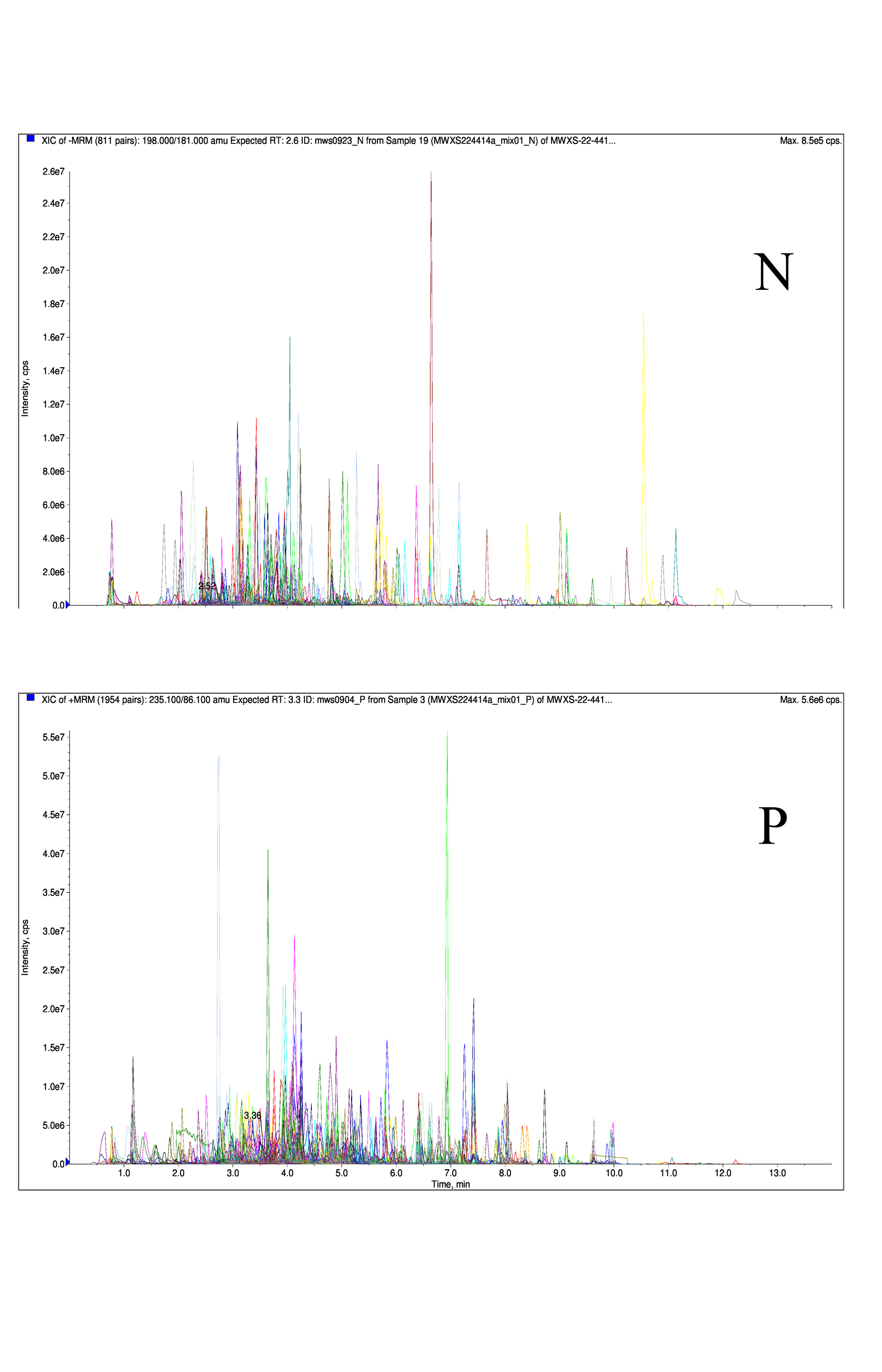


**Fig. S3.** Multiple reaction monitoring (MRM) graph of the QC sample. Note: Each color indicates a detected metabolite in the sample. The abscissa is the retention time of the metabolite. The ordinate is the ion current intensity of the ion detection (the intensity units are counts per second (cps)). N stands for negative ion mode; P stands for positive ion mode.


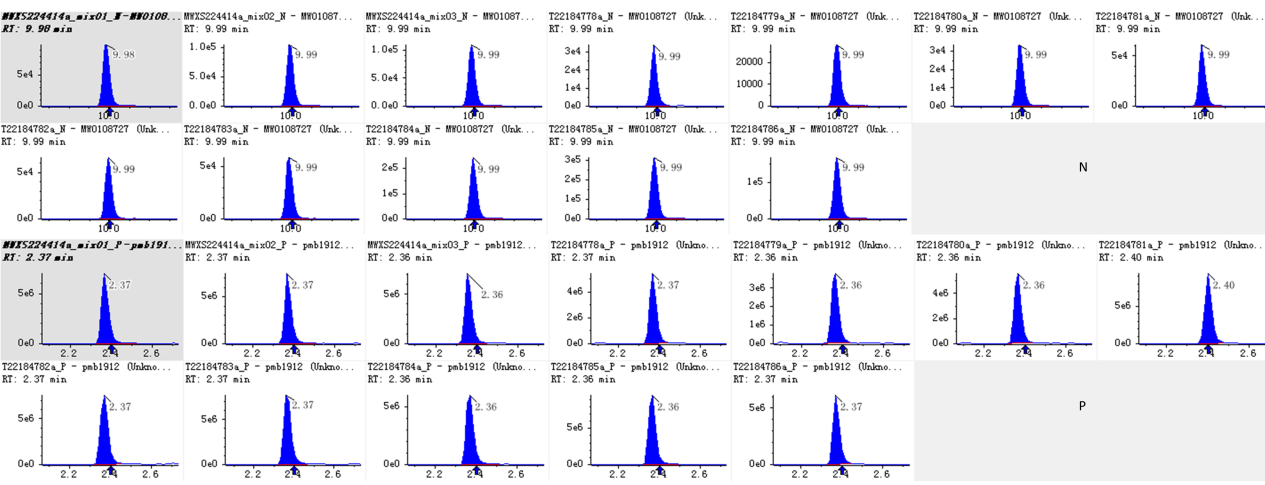


**Fig. S4.** Integral correction diagram for quantitative analysis of some of the detected metabolites. The abscissa is the retention time (min) of the metabolite detection. The ordinate is the ion flow strength (CPS) detected by a metabolite ion. The peak area represents the relative content of the substance in the sample.


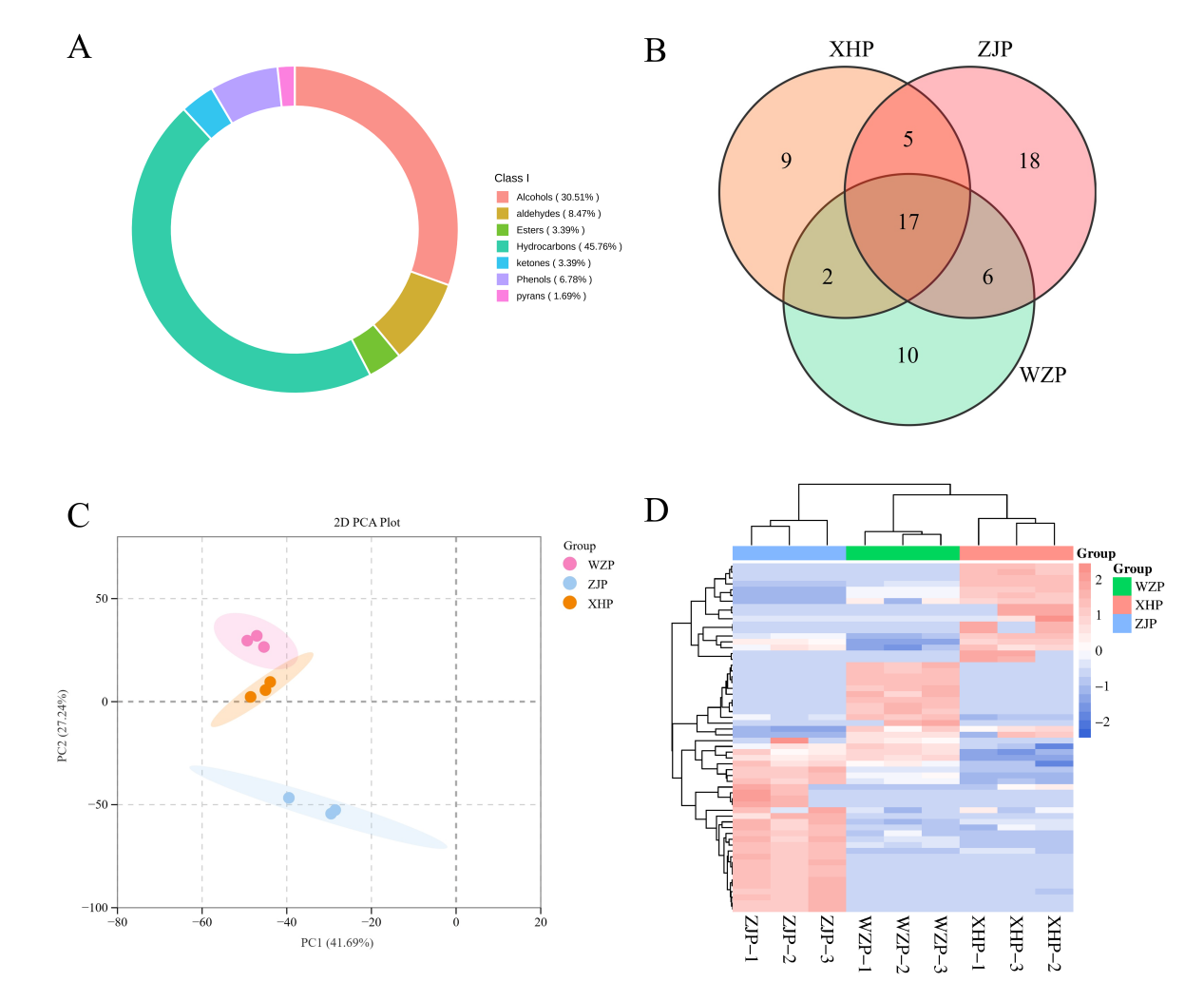


Fig. S5. VOCs profiles and differences between ZJP and WZP and XHP. (A) Pie chart of the classification of identified VOCs. (B) Distribution of VOCs. (C) PCA. (D) Clustering heat map of VOCs.


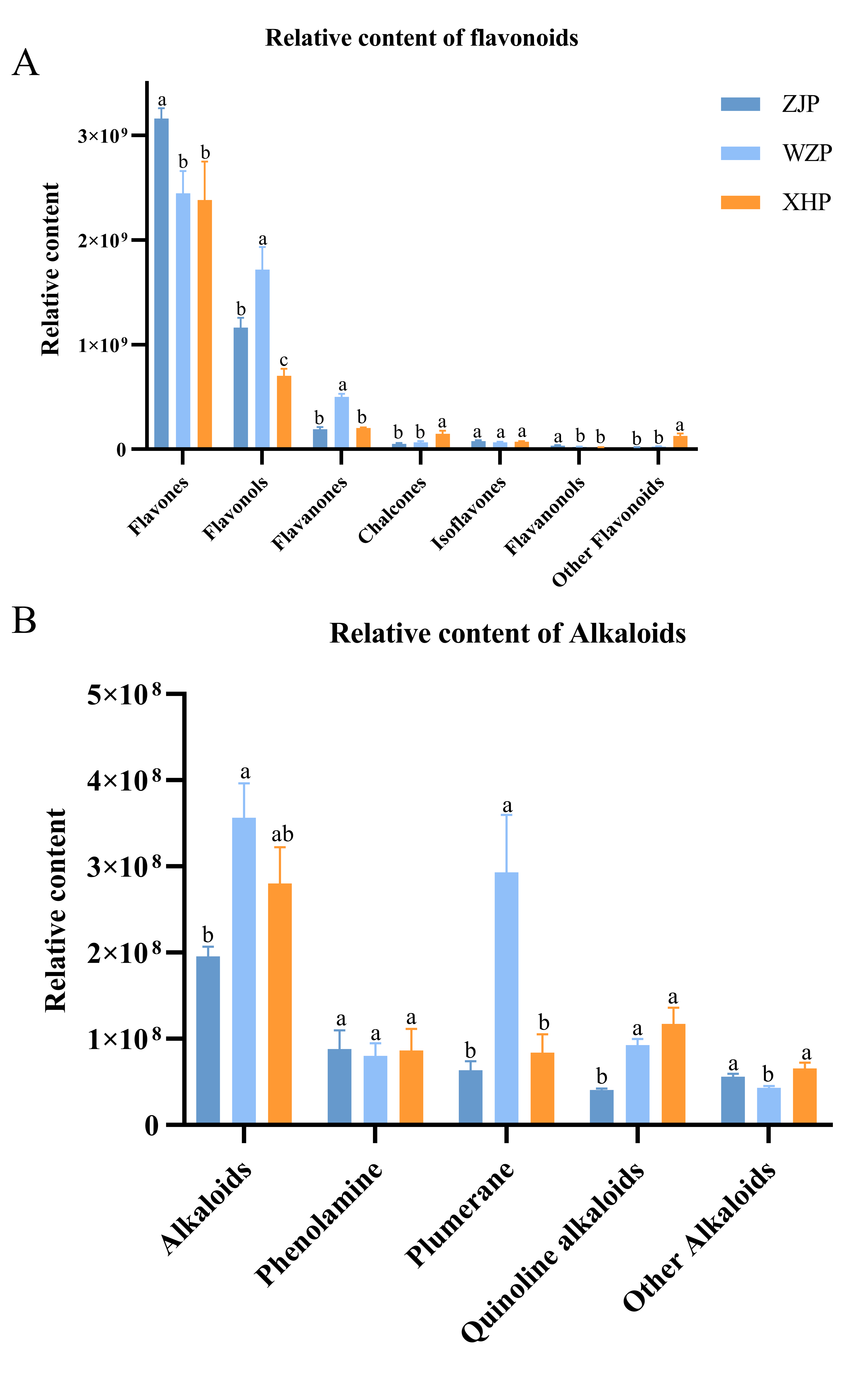


**Fig. S6.** Relative contents of flavonoids and alkaloids: (A) Relative content of flavonoids; (B) Relative content of alkaloids. Different letters in the graphs indicate significant differences in the same index (P < 0.05).


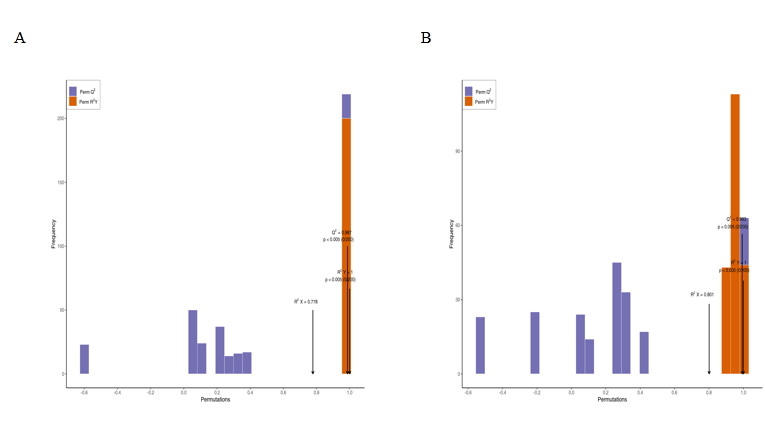


**Fig. S7.** OPLS-DA verification diagram of the pairwise comparison of metabolites: (A) WZP vs ZJP; (B) XHP vs ZJP.


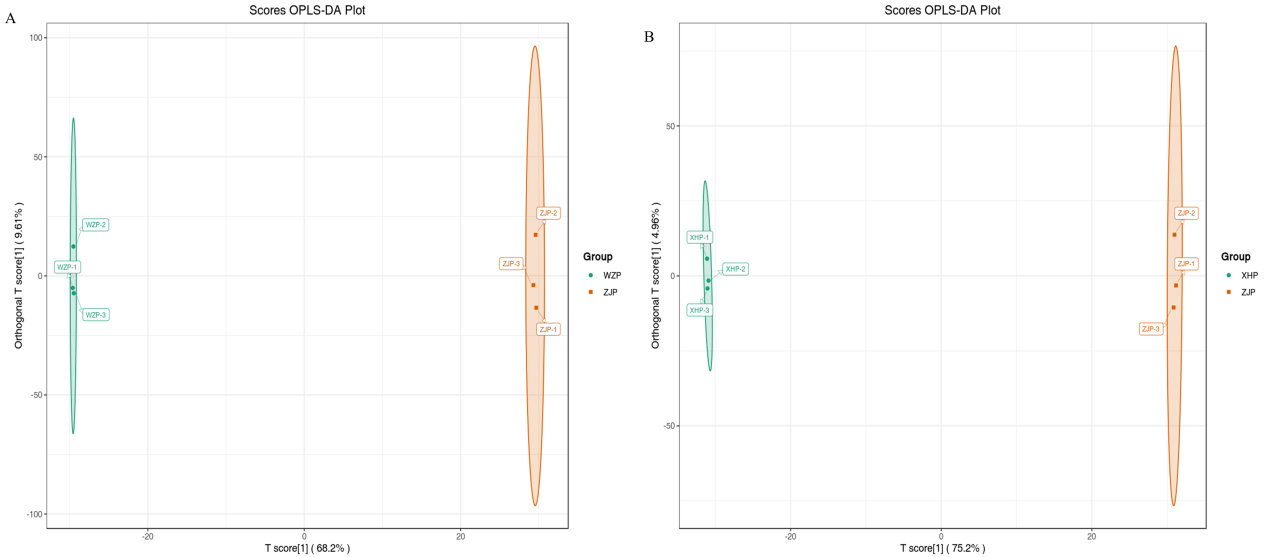


**Fig. S8.** The score plots of OPLS-DA pairwise comparisons of metabolites: (A) WZP vs ZJP; (B) XHP vs ZJP.

**
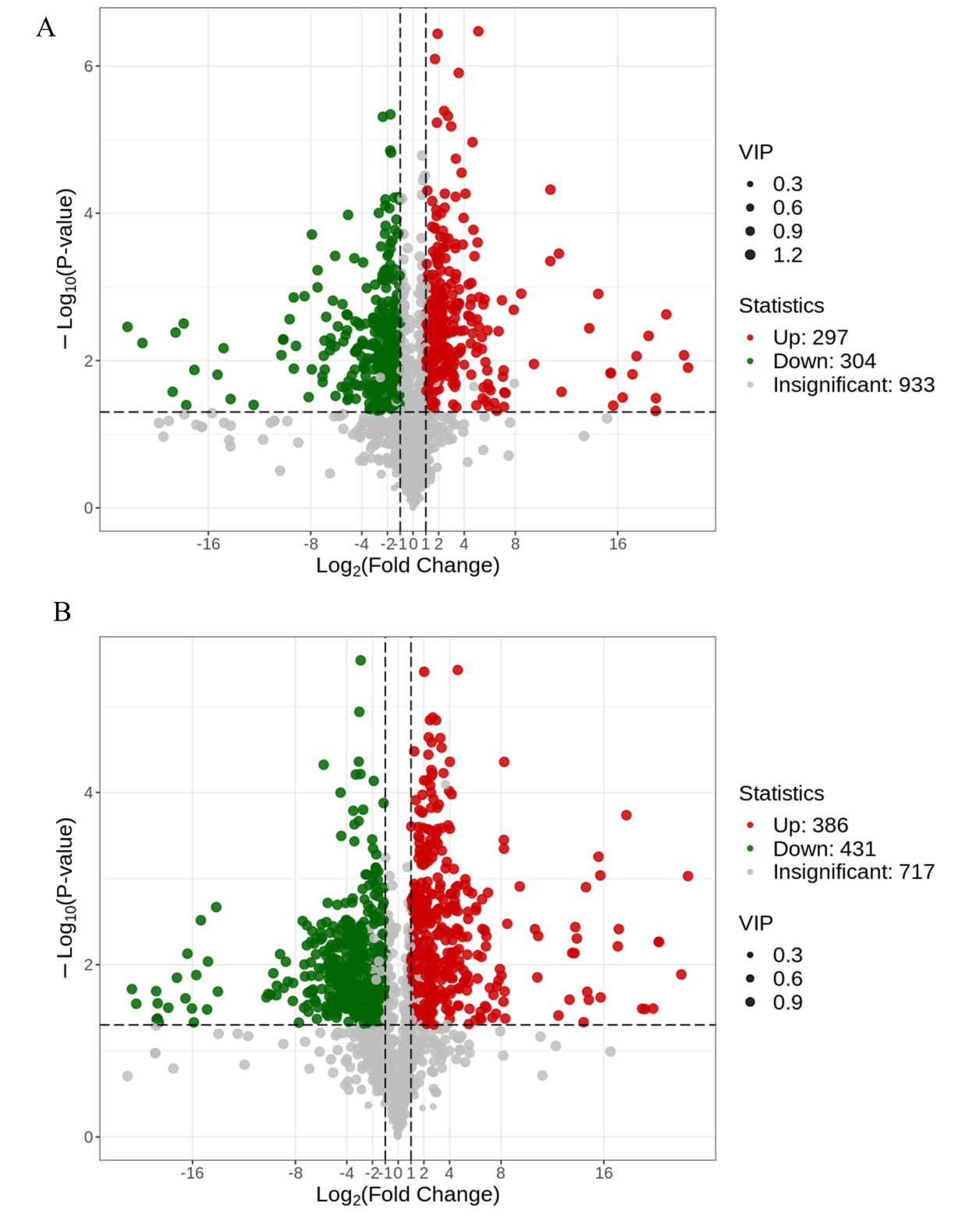
**

**Fig. S9.** Volcano plots of differential metabolites in the pairwise comparison: (A) WZP vs ZJP; (B) XHP vs ZJP.. Note: Each point in the volcano map represents a metabolite, the abscissa represents the logarithmic value of the difference of relative content of a certain metabolite in the two samples, the ordinate represents the VIP value. The greater the absolute value of the abscissa, the greater the multiple difference in the expression level between the two samples; the greater the ordinate value, the more significant the differential expression, and the more reliable the differentially expressed metabolites screened. In the figure, the green dots represent down-regulated differentially metabolites, the red dots represent up-regulated differentially metabolites, and gray represents detected but not significantly different metabolites.


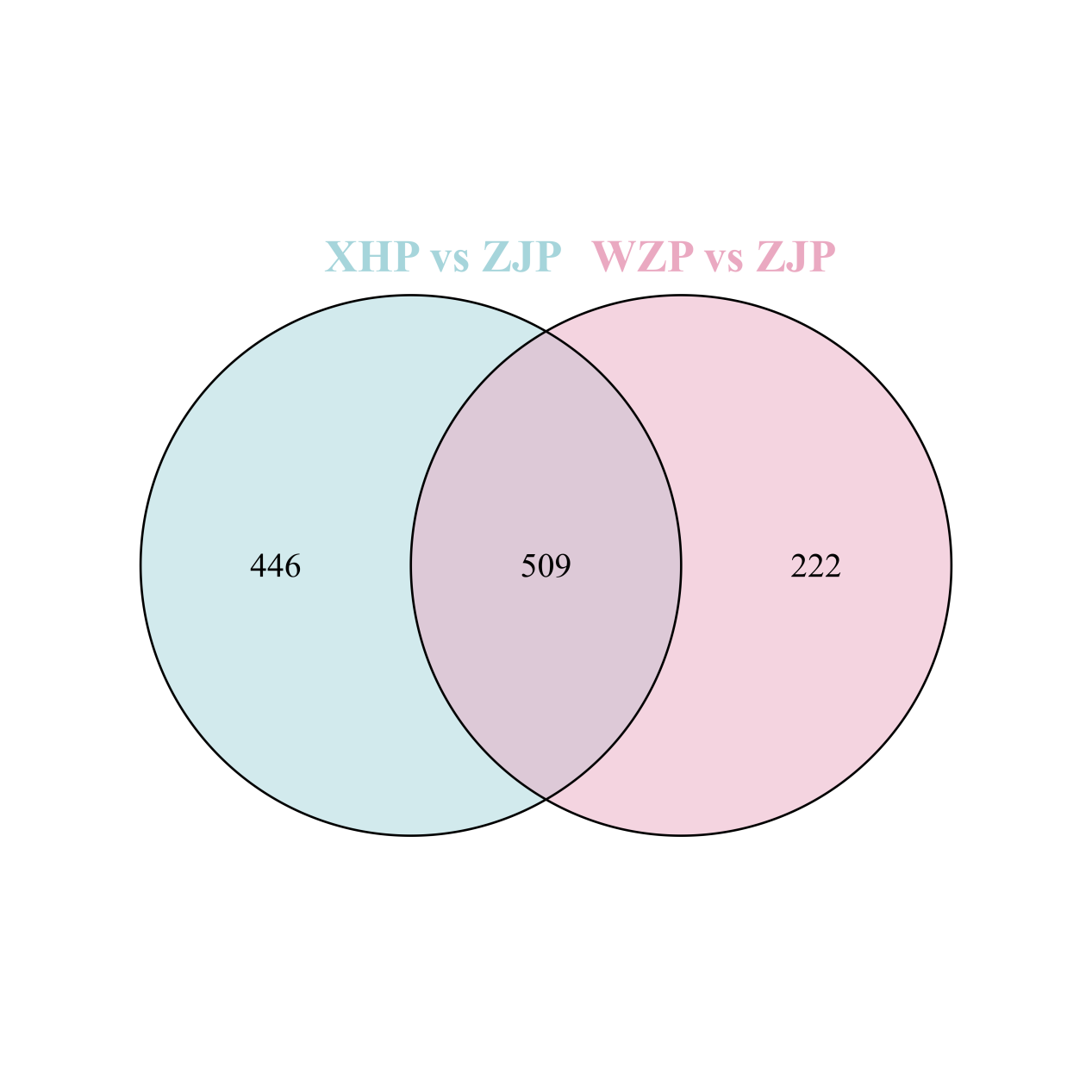


**Fig. S10.** WZP vs ZJP and XHP vs ZJP common DMs.


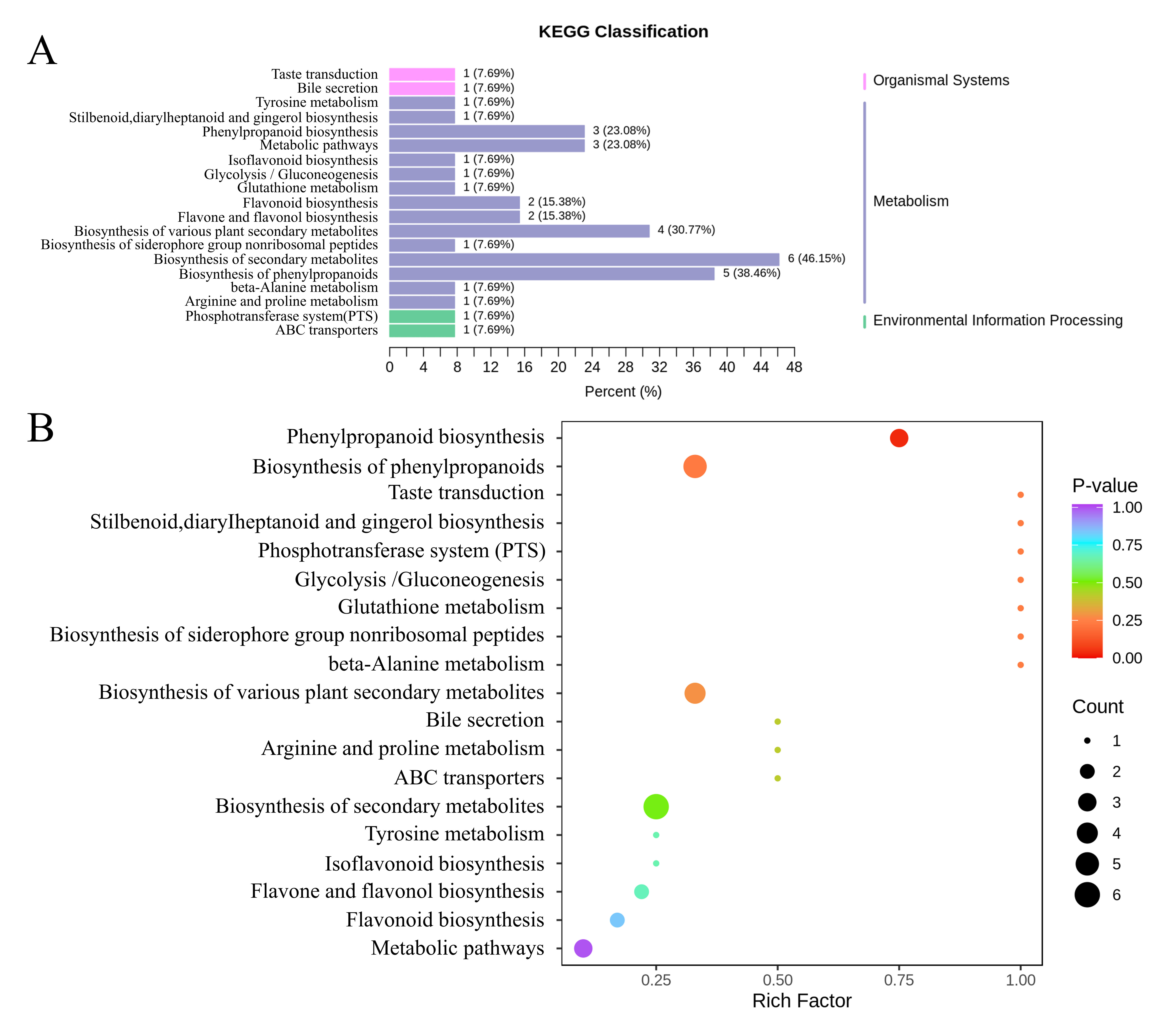


**Fig. S11.**  Key DMs KEGG analysis: (A) Key DMs KEGG pathway annotation. (B) Key DMs KEGG enrichment analysis. KEGG enrichment plot horizontal coordinate indicates the corresponding Rich Factor of each pathway, vertical coordinate is the pathway name (sorted by P-value), and the color of the dots reflects the size of the P-value, with the redder indicating the more significant enrichment. The size of the dots represents the number of DMs enriched.
